# A label-free G-quadruplex aptamer/gold nanoparticle-based colorimetric biosensor for rapid detection of bovine viral diarrhea virus genotype 1

**DOI:** 10.1371/journal.pone.0293561

**Published:** 2024-07-30

**Authors:** Parisa Rabiei, Hassan Mohabatkar, Mandana Behbahani

**Affiliations:** Department of Biotechnology, Faculty of Biological Science and Technology, University of Isfahan, Isfahan, Iran; Consiglio Nazionale delle Ricerche, ITALY

## Abstract

Bovine viral diarrhea virus (BVDV) is the cause of bovine viral diarrhea disease, one of the most economically important livestock diseases worldwide. The majority of BVD disease control programs rely on the detection and then elimination of persistent infection (PI) cattle, as the continuing source of disease. The main purpose of this study was to design and develop an accurate G-quadruplex-based aptasensor for rapid and simple detection of BVDV-1. In this work, we utilized *in silico* techniques to design a G-quadruplex aptamer specific for the detection of BVDV-1. Also, the rationally designed aptamer was validated experimentally and was used for developing a colorimetric biosensor based on an aptamer-gold nanoparticle system. Firstly, a pool of G-quadruplex forming ssDNA sequences was constructed. Then, based on the stability score in secondary and tertiary structures and molecular docking score, an aptamer (Apt31) was selected. In the experimental part, gold nanoparticles (AuNPs) with an average particle size of 31.7 nm were synthesized and electrostatically linked with the Apt31. The colorimetric test showed that salt-induced color change of AuNPs from red to purple-blue occurs only in the presence of BVDV-Apt31 complex, after 20 min. These results approved the specificity of Apt31 for BVDV. Furthermore, our biosensor could detect the virus at as low as 0.27 copies/ml, which is an acceptable value in comparison to the qPCR method. The specificity of the aptasensor was confirmed through cross-reactivity testing, while its selectivity was confirmed through plasma testing. The sample analysis showed 90% precision and 94% accuracy. It was concluded that the biosensor was adequately sensitive and specific for the detection of BVDV in plasma samples and could be used as a simple and rapid method on the farm.

## 1. Introduction

Bovine viral diarrhea virus (BVDV), the cause of bovine viral diarrhea (BVD) disease, belongs to the family *Flaviviridae* genus *Pestivirus* [1]. Among 11 different species of *Pestivirus* (A-K), species A and B, known as BVDV-1 and BVDV-2 respectively, are the most important [2]. The BVDV contains a positive-sense single-stranded RNA (12.3–16.5 kb) encoding four structural and eight non-structural proteins [3–5]. Structural proteins of BVDV are nucleocapsid protein C, Erns, E1, and E2 glycoproteins which the last three are anchored in the virus envelope. Glycoprotein E2, the largest and the most exposed protein on the virus envelope surface, is vital for interaction with the host cell and virus entry [6, 7]. Commonly reported symptoms of BVDV are diarrhea, immunosuppression, abortion, growth retardation, decreased fertility, and reduced milk production. In addition, infection of the fetus during the 40 to 150 days of the pregnancy can result in the formation of persistently infected (PI) calves which are the main source of BVDV spread in the herd. According to clinical investigations, PI cattle exhibit a normal range of heart rate and body temperature. However, they suffer from lower feed intake, lower concentration of thyroid hormones, and growth retardation [3, 8, 9]. Because of having serious clinical symptoms in a broad range of host animals, including cattle, sheep, pigs, goats, buffalo, and camels [10], BVD disease is considered as one of the most economically important diseases in the livestock industry [11]. Besides meat and milk reduction costs, the main costs of BVD disease include vaccinations, diagnosis of PI animals, and their treatments or replacement [12, 13]. Because of different farming systems and control programs, BVD disease prevalence varies across regions. Based on statistical reports, antibody prevalence is 51–77% in sub-Saharan Africa and 46% in Europe, while antigen prevalence is approximately 19% and 0.2%, respectively. The financial losses and epidemiology effects of BVDV infection are well-quantified in high-income countries. In beef herds in North America, infection with BVDV leading to calf mortality can cost up to US$88/animal [14].

The majority of BVDV control and eradication programs are established based on two fundamentals, vaccination and diagnosis, especially the diagnosis of PI animals [15]. Various techniques are implemented for the detection of acute or persistent BVDV infections. These techniques include virus isolation, PCR-based methods, ELISAs and more recent techniques such as loop-mediated isothermal amplification (LAMP), dot-blot assay [2, 16–18]. Since these methods are laborious, time and cost-consuming, developing new accurate and rapid detection techniques such as biosensors, as a new generation of sensing technology, is essential. A biosensor is an analytical device that provides a fast, sensitive, and specific detection of biomaterial samples by converting a biological response into an observable or measurable signal. Compared with different types of biosensors, colorimetric ones have some obvious advantages, such as easy fabrication, low cost, quick detection, high sensitivity and selectivity, and qualitatively/semi-qualitatively easy naked-eye sensing [19, 20]. Various types of bio-recognition elements are available to allow biosensor platforms to be tailored for specific detection, including antibodies, peptides, and aptamers [21].

Aptamers refer to synthetic short nucleic acid sequences (RNA, single-stranded DNA, or modified versions) that adopt a distinctive structure, enabling them to selectively and strongly attach to particular substances like amino acids, medications, viruses, proteins, as well as various other molecules. They can also attach to cells, tissues, and even entire organisms [22, 23]. Moreover, G-quadruplex aptamers show more thermodynamic and biological stability compared to the other aptamers [24]. The process of selecting aptamers through in vitro methods is laborious, time-intensive, and frequently does not result in the identification of aptamers with strong binding affinities [25]. Accordingly, numerous studies are carried out using bioinformatics for designing an optimized aptamer with a high affinity for the target [26].

In this work, we aimed to develop and validate a rapid, simple G-quadruplex-based aptasensor for the diagnosis of BVDV-1 in very low concentration, for the first time. To this aim, we designed a G-quadruplex aptamer via bioinformatics methods. Next, the experimental assessment was conducted to determine the precise attachment of the designed aptamer. This aptamer was then employed to create a diagnostic tool, an aptasensor based on color-changing gold nanoparticles, intended for the detection of BVDV-1. So far, no G-quadruplex-based aptasensor has been reported for detecting BVDV-1. Detection of very low amounts of the virus in samples, highly specific binding, a short detection time, and facilitated target detection are advantages of our developed BVDV aptasensor [27–29].

## 2. Materials and methods

### 2.1. *In silico* selection, optimization, and design of BVDV-binding aptamer

The rational design of an aptamer requires several steps as explained in details below.

#### 2.1.1. Protein data collection and preparation

The coordinate file of the E2 protein with the code 4JNT was obtained from the RCSB Protein Data Bank (PDB). Three different entries are accessible for BVDV-1 E2 protein: 4JNT, 2YQ2, and 2YQ3 in various pH values, 7.5, 8 and 5, respectively [30]. 4JNT was selected because of its reported pH proximity to bovine blood pH [31]. Since the structure resolution of 4JNT is not high (4.09Å), an energy minimization step was done via YASARA server (http://www.yasara.org/minimizationserver.htm) before performing molecular docking. Energy minimization can refine the side chain placement and improve the physical realism and stereochemistry of the entry. Water and miscellaneous ligand molecules in the coordinate file of the E2 protein were removed, as well. For specific detection of the BVD-1 viruses based on E2 protein, it is necessary to find particular amino acids which are conserved among BVDV-1 strains. The sequences of BVDV-1 E2 protein were fetched from the NCBI database (https://www.ncbi.nlm.nih.gov) and multiple sequence alignment analysis was performed by the T-Coffee algorithm (http://tcoffee.crg.cat).

In order to validate the specific binding of the designed aptamer, the BSA was selected as the control protein in the molecular docking. The crystal structure of BSA with the entry code 4F5S was downloaded from the PDB website. Steps of structure preparation were applied on the 4F5S similar to what was performed for 4JNT.

#### 2.1.2. G-quadruplex ssRNA library preparation

A library consisting of 100 G-quadruplex ssRNA sequences with different lengths of up to 32 nucleotides was built based on several experimentally validated G-quadruplex sequences described in Nucleic acid G-quadruplex Structure Interacting Proteins DataBase (G4IPDB) [32] as the reference sequences. Deletion, insertion, and point mutation changes were implemented on the reference sequences to build the G-quadruplex ssRNA library. The RNA sequences included a) four repeats of binary, ternary, and quadruplet G bases, and b) loops with 1–5 random bases. The probability of G-quadruplex formation of the library sequences was analyzed via QGRS Mapper software (https://bioinformatics.ramapo.edu/QGRS/index.php) [33]. The sequences which had a QGRS score of more than 60 were selected for further analysis.

#### 2.1.3. G- quadruplex ssRNA structure prediction

In this study, QGRS mapper-validated sequences were selected and analyzed for predicting their secondary structure by RNAfold web server. The following options were chosen: a) fold algorithm: minimum free energy (MFE) and partition function, b) energy parameters: RNA parameters, c) the desired temperature for rescaling the energy parameters: 25°C. In addition, the option “incorporate G–Quadruplex formation into the structure prediction algorithm” was selected.

*In silico* modeling of the tertiary structure of RNA/DNA molecules is one of the challenging topics in computational biophysics, especially for structures with non–Watson-Crick hydrogen bonds such as quadruplexes [34, 35]. In this study, Rosetta FARFAR (RNA De Novo) Protocol (https://rosie.rosettacommons.org/rna_denovo) [36, 37] was used to predict the tertiary structure of the aptamers. The sequence order of each ssRNA aptamer in the library was input to the server and the 3D structures were obtained after 50000 Monte Carlo cycles.

To achieve DNA aptamers, the obtained tertiary structures of RNA aptamers were converted to DNA via Discovery Studio 2020 Client v.20.1.0. Afterward, a geometry optimization was applied to DNA aptamers through the Steepest Descent algorithm in 200 cycles via HyperChem Professional program.

#### 2.1.4. Docking of ssDNA aptamers with Bovine-E2 protein

Molecular docking was performed by HDOCK and PatchDock servers [38]. The prepared BVDV-1 E2 protein structure (explained in section 2.1.1) and DNA aptamers’ tertiary structure were input to the server to perform blind DNA-protein docking. Then, the results were sorted based on docking scores. The complexes with high dock scores in both docking servers were analyzed in the next step via the LIGPLOT program [39] to generate a 2D diagram of protein-DNA interaction. Thereafter, the critical step was to interpret the results of the LIGPLOT program and select the aptamers which bind to desired amino acids, described in section 2.1, and form strong enough hydrogen bonds and hydrophobic contacts. In this step of screening, a small number of aptamers were eligible to be selected. Then, one more molecular docking analysis was performed on selected aptamers with BSA protein, as a negative control, to find an aptamer that shows far less affinity for BSA.

### 2.2. Design and fabrication of aptasensor

#### 2.2.1 Reagents, materials, and equipment

The designed aptamer was synthesized by TAG Copenhagen A/S Company, Denmark. qPCR primers were received from SinaColon Co. Iran. The cDNA synthesis kit and SYBR^™^ Green PCR Master Mix were purchased from AddBio Inc. (Korea) and Biotechrabbit (Germany), respectively. BehPrep Viral Nucleic Acid Extraction Kit was obtained from BehGene Biotechnology Company, Iran. Chloroauric acid (HAuCl_4_.3H_2_O) was obtained from Sigma-Aldrich (United States). Trisodium citrate, ethanol, methanol, potassium chloride, nitric acid, and hydrochloric acid were prepared from Merck Company (Germany). UV/Vis spectrophotometer (Agilent 8453, Agilent Technologies, Inc., US), Nanodrop (OneC, Thermo, US), Fluorescence spectroscopy (BioTek Cytation5 multi-mode reader), Real-Time PCR Systems (StepOne^™^, Applied Biosystems, US) and Dynamic light scattering (VASCO / Cordouan Technologies / France), and Zeta Potential analyzer (SZ-100/Horiba Scientific/Japan).

In addition, four BVDV-infected (PI animal) and two healthy cattle blood samples were fetched from an industrial dairy cattle farm in Isfahan province in Iran. Blood samples were collected by heparin venojects and plasma was separated using centrifugation at 4000 rpm for 10 minutes.

#### 2.2.2. Aptamer specification and G-quadruplex forming analysis

In order to assess the G-quadruplex structure forming of Apt31 (the selected aptamer) and determine the specific binding of aptamer into BVDVs, a label-free technique using the crystal violet (CV) and fluorescence spectrophotometer was applied.

All fluorescent measurements were executed in a 96-well plate. The 2.5μM solution of G-quadruplex DNA was prepared in 25 mM Tris-HCl buffer (pH 7). A CV solution with a concentration of 5 μM was used. The test was performed in four conditions, 1) Tris-HCl buffer and 10 μl of CV (final concentration of 0.25 μM), 2) Tris-HCl buffer, 10 μl of CV and KCl (final concentration of 1mM), and 3) Tris-HCl buffer, 10 μl of CV, 10 μl of aptamer solution (final concentration of 0.125 μM) and KCl. 4) Tris-HCl buffer, 10 μl of CV, 10 μl of aptamer solution and KCl, and four different concentrations of BVDV- infected plasma (1:10, 1:100, 1:1000, 1:10000) and one healthy plasma (as negative control). In addition, a random non-quadruplex aptamer was used as control. The CV was excited at wavelength of 580 nm, and the emitted fluorescence was detected in the range of 600–700 nm. The experiment was repeated three times.

#### 2.2.3. Gold nanoparticle synthesis

Spherical gold nanoparticles were synthesized by citrate reduction of chloroauric acid based on the Turkevich method [40]. The solutions of 1% HAuCl_4_ and 1% Trisodium citrate solutions were made separately. The solution of HNO_3_–HCl (1: 3 v/v) was used to clean all glassware before use. Briefly, 100 μl of 1% HAuCl_4_ solution was added to 10 ml deionized water in an Erlenmeyer flask on a hot stirring plate. Once the HAuCl_4_ solution starts boiling, 600 μl of trisodium citrate solution 1%, as a reducing and stabilizing agent, was added quickly. The solution was kept vigorously stirring for 15 min until the color changed from yellow to wine red. After that, the red solution was transferred to an ice-containing dish and continued to stir for 15 min. The formation of AuNPs was confirmed by a UV-vis spectrometer. The nanoparticles were characterized by the Zetasizer instrument and Transmission electron microscopy (TEM).

#### 2.2.4. Salt tolerance test of gold nanoparticles

Salt-induced aggregation of gold nanoparticles (Au-NP) was evaluated in different concentrations of KCl solution. To perform this test, 10 μl of KCl with concentrations of 200, 400, 500, 600, 800 mM, and 1M was added to 200 μl of Au-NP solution, separately. As the KCl solution was diluted 1:21 in test tubes, the final concentration was 9.5, 19, 23.8, 28.5, 38, and 47.6 mM, respectively. Color change of solutions was monitored after 15 min by eye-naked and then with a UV-vis spectrometer. The minimum concentration of KCl in which the color of the AuNP solution changed to purple was selected for further experiments. The experiment was repeated in triple.

#### 2.2.5. Optimization of aptamer concentration and performing colorimetric assay to determine aptamer sensitivity and specificity

The selected concentration of KCl in the previous step was used for performing the colorimetric test. This test was done in two stages: 1) the minimum amount of aptamer which can prevent AuNPs from aggregation in the presence of KCl was detected, and 2) The minimum copy number of the virus which can be detected by the aptamer-AuNP system was found. In the first stage, 2, 4, 6, 8, and 10μl of DNA aptamer with final concentrations of 0.47, 0.93, 1.38, 1.83, and 2.27 μM, respectively, was added to 200 μl of AuNP solution. After 5 min, 10 μl of KCl solution was added and the color change of Au-NPs was monitored. To evaluate the electrostatic binding of aptamer on gold nanoparticle surface, Dynamic light Scattering (DLS), zetasizer and Fourier transform infrared (FTIR) analysis were performed for non-functional and functionalized AuNPs.

In the second stage, the optimized amount of aptamer solution was added to the 200μl of AuNP solution. After 5 min, 10 μl of diluted BVDV-infected plasma solutions with the range of 10^11^, 10^8,^ 10^5,^ 10^3^, 10^1,^ and 10^−1^ virus copies/ml were added. After 5 min, 10μl of optimized KCl solution was added. In addition, four vials containing a) virus-negative plasma, b) bovine leukemia virus (BLV)-infected plasma, c) AuNP+ aptamer solution (without virus), and d) Au-NP solution were tested as controls, respectively. The color change from red to purple was observed visually after 15–20 min and the absorbance of samples at the wavelength of 400 to 700 nm was measured via a multimode microplate reader. All experiments were repeated three times. In addition, field emission scanning electron microscope (FESEM) characterization was performed for three samples: a) AuNPs, b) AuNPs+Apt31+KCl, and c) AuNPs+Apt31+BVDV+KCl.

#### 2.2.6. Sample testing and data analysis

To assess the analytical features of the aptasensor, fifty plasma samples with confirmed infection status through RT-PCR were obtained from specialized veterinary laboratories. The set comprised thirty BVDV-infected samples and twenty negative samples. All samples were diluted at a ratio of 1:10^−4^ in distilled water to prevent interference of viruses in AuNPs aggregation. The accuracy and precision of the aptasensor were evaluated qualitatively through visual observation of the color change in the AuNPs. The formulas used for assessment are elaborated in detail in the references [41, 42].

### 2.3. Detection and quantification of BVDV via real-time PCR

#### 2.3.1. Virus RNA genome extraction

The virus RNA genome of prepared samples was extracted by the Behgene^™^ kit method as follows: 200 μl of each plasma sample was added to a microtube. Thereafter, 300 μl of lysis buffer containing RNA carrier was added and then vortexed for 15 seconds until a homogenous solution was achieved. RNA carrier can promote the binding of RNA to silica membrane and prevents RNA degradation. Afterward, the sample microtube was incubated at room temperature for 15 minutes. 250 μl of 96% ethanol was added to each microtube, followed by 15 seconds of vortexing. To extract the RNA, all content of the lysate prepared in the previous steps was transferred to the center of the spin column. The column was centrifuged at 8000 rpm for 1 minute. The washing phase was done in two steps by adding 400 μl of washing buffer I and II and then, centrifugation at 8000 and 13500 rpm for 1 and 5 minutes, respectively. Finally, 150 μl of elution buffer was added to the center of the column. After given 5 minutes, the collection tube was centrifuged at 8000 rpm for 1 minute. The permeate contained RNA. The concentration of extracted RNA samples was measured using Nanodrop Spectrophotometers (Thermo Scientific ^™^, Model One C). In addition, the quality of ssRNA was evaluated by 1% agarose gel electrophoresis in TAE buffer.

#### 2.3.2. Complementary DNA synthesis

Complementary DNA (cDNA) was synthesized via AddScript cDNA Synthesis Kit reagents. The reaction was performed in a total volume of 20 μl containing 10 μl of 2x reaction buffer, 2 μl dNTP mixture (10mM), 2 μl random hexamer primer, and 150 ng RNA sample. Deionized water was added to bring to the final volume. The temperature cycle was performed as follows: 25 °C for 10 min, 50 °C for 60 min, 80 °C for 5 min, and then, 12 °C as holding temperature.

#### 2.3.3. Performing real-time PCR analysis

Quantitative PCR (qPCR) or real-time PCR as an advanced standard technique for the detection and measurement of the BVDVs was performed. The sequence of the forward and reverse primers used in this work were 5’GGTAGCAACAGTGGTGAG3’ and 5’GTAGCAATACAGTGGGCC3’, respectively. The primers were selected from Anita et al. study [43]. RT-PCR amplification was carried out in a total volume of 11 μl including 2 μl of prepared cDNA, 2.75 μl of SYBR Green 4X master mix, 0.4 μl from each forward and reverse primer, 5.45 μl of distilled water, in a condition of 3 min at 95 °C, 45 cycles of 10 sec at 95 °C, 10 sec at 59 °C (as annealing temperature) and 15 sec at 72 °C, and then 15 sec at 95 °C and 1 min at 60 °C.

### 2.4. Statistical assay

IBM SPSS Statistics program was used for statistical analysis. Data groups were compared via Tukey’s one way ANOVA method. Values with *p* < 0.05 (*) were considered statistically important.

## 3. Results and discussion

### 3.1. *In Silico* design of BVDV-binding aptamer

#### 3.1.1. G-quadruplex ssRNA library preparation and aptamer structure prediction

Over the past decades, several algorithms have been developed to predict RNA and DNA secondary and tertiary structures from their sequences. Among several methods for the prediction of RNA/DNA secondary structure, it is distinguished that the RNAfold algorithms can deal with quadruplex structures more accurately [44]. In the first step, sequences in the G-quadruplex ssRNA library were narrowed down based on the QGRS mapper score. Considering this score, 55 sequences that obtained a score > 60 were chosen for further analysis. In the second step, the minimum free energy of the aptamers’ secondary structure reported by RNAfold as a stability index was considered. Thirty-three sequences showed a minimum free energy of less than -30 kcal/mol. These sequences were selected for tertiary structure prediction and docking analysis. The best 10 sequences are shown in Table 1.

**Table 1:** The aptamer sequences with the highest QGRS score, their GC-content percentage, and the RNAFold energy results.

Aptamer Name	Sequence	GC%	QGRS Score	RNAFold MFE*
Apt22	GGGGTATCGGGGTTCGGGGAGGGGATCGGGG	74.2	61	-47.41
Apt23	GGGGTGGGGAGGGGTCGGGGATGGGGAGGGG	80.6	92	-57.77
Apt25	GGGGTGGGGAGGGGTCGGGGATCGGGGCCCC	83.9	62	-58.39
Apt26	GGGGTGGGGAGGGGTTGGGGATGGGGACCCC	77.4	92	-63.43
Apt27	GGGGTGGGGACGGGGTGGGGATGGGGACCCC	80.6	92	-63.43
Apt28	ATGGGGAGTGGGGAGTGGGGAGTGGGGAGTGG	68.8	63	-43.32
Apt31	GGGGTATCGGGGATGGGGAGGGGATCGGGG	73.3	61	-49.77
Apt32	GGATCGGGGATGGGGATGGGGATCGGGGGATC	68.8	62	-49.41
Apt33	GATCGGGGATGGGGATGGGGATCGGGGGATCC	68.8	68	-50.16
Apt34	CTGGGGATGGGGCTGGGGAAGGGGTAGGGGAC	71.9	69	-37.86

* Minimum Free Energy (MFE)

#### 3.1.2. Designed aptamer structure and docking score

The E2 protein is made up of four domains, DA (residues 1–87), DB (residues 88–164), DC (residues 165–271), and DD (residues 272–333) [7]. Domains DA and DB containing Ig-like domain, are the most exposed domains in the tertiary structure of the protein and are responsible for the entry of the virus to the host cell and binding to neutralizing antibodies as well [7]. Four N-linked glycosylation sites have been reported in E2 protein including residues Asp117 located in domain DB, Asp186 and Asp230 in domain DC and Asp 298 in domain DD [45]. Subsequently, the non-glycosylated domain DA and several non-glycosylated regions in DB were selected. Evaluating three screening parameters: a) most exposed region, b) non-glycosylated regions and c) most conserved amino acids, residues 733–784 were chosen as the appropriate region for binding of the designed aptamer.

Molecular docking analysis was performed for 33 aptamer sequences selected in previous steps to investigate their binding tendency and orientation to BVDV-1 E2 protein. The five aptamer sequences which resulted in the best docking scores are shown in Table 2. Among them, Apt31 was chosen as the final aptamer because of its desired binding site to BVDV-1 E2 protein and appropriate tertiary structure, and high docking score.

**Table 2:** Docking score and binding site of best five aptamers obtained in HDOCK and PatchDock servers.

Aptamer Name	Binding site (Residue Number)	Score	
		HDock	PatchDock
Apt22	900–1020	-267.05	14226
Apt25	739–858	-274.24	14160
Apt27	752–923	-288.53	15716
**Apt31**	**739–792**	**-270.48**	**14034**
Apt32	753–850	-293.19	13216

**Fig 1:**
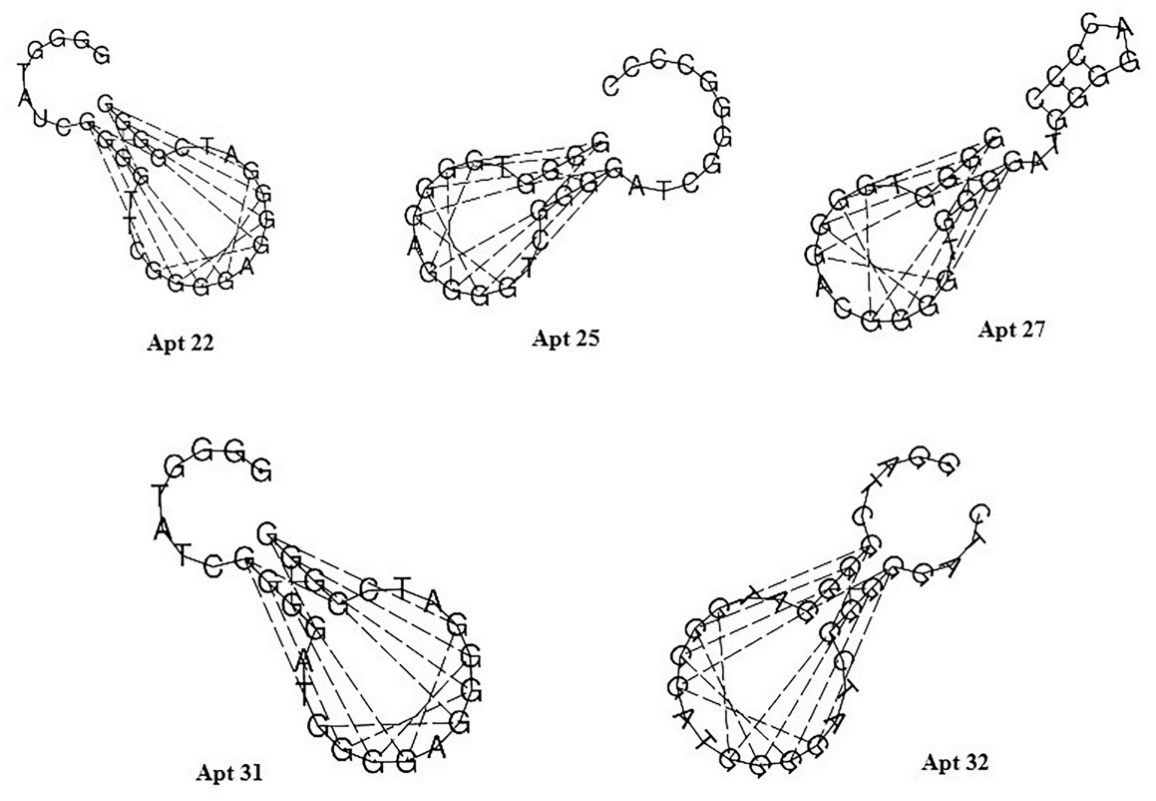
Secondary structure of five aptamers with the highest docking scores. Structures are predicted by the RNAfold web server. Dash lines indicate G-G interactions responsible for forming of G-quadruplex structure.

**Fig 2:**
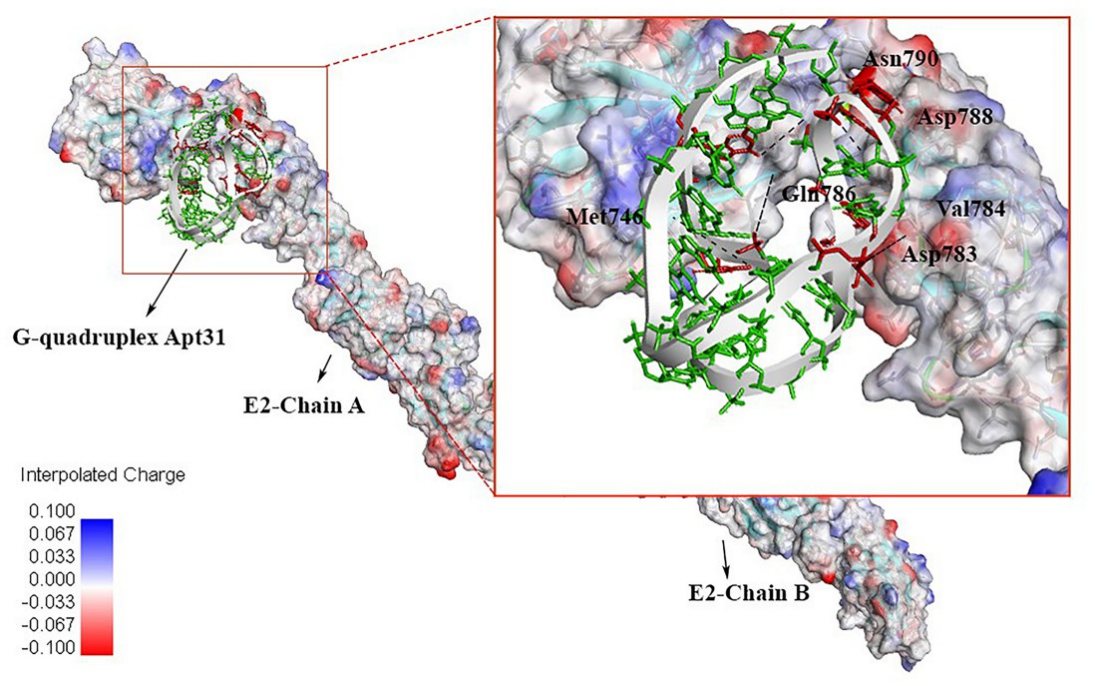
Interaction of Apt31 with the BVDV-E2 protein (chain A and B) with respect to interpolated charge surface representation. H-bonding amino acids are shown in the magnified part. Interacting aptamer nucleotides are colored in red, and dashed black lines represent H-bonds.

The secondary structure of the mentioned five aptamers obtained from the RNAfold web server is shown in Fig 1. Minimum free energy of -49.77 kcal/ mol was achieved in the secondary structure analysis of DNA Apt31. The tertiary structure of Apt31 (RNA backbone) was predicted by the Rosetta FARFAR server and then underwent some changes including RNA to DNA backbone conversion and geometry optimization. The tertiary structure of Apt31 (DNA backbone) interacting with the E2 protein is shown in Fig 2.

**Fig 3:**
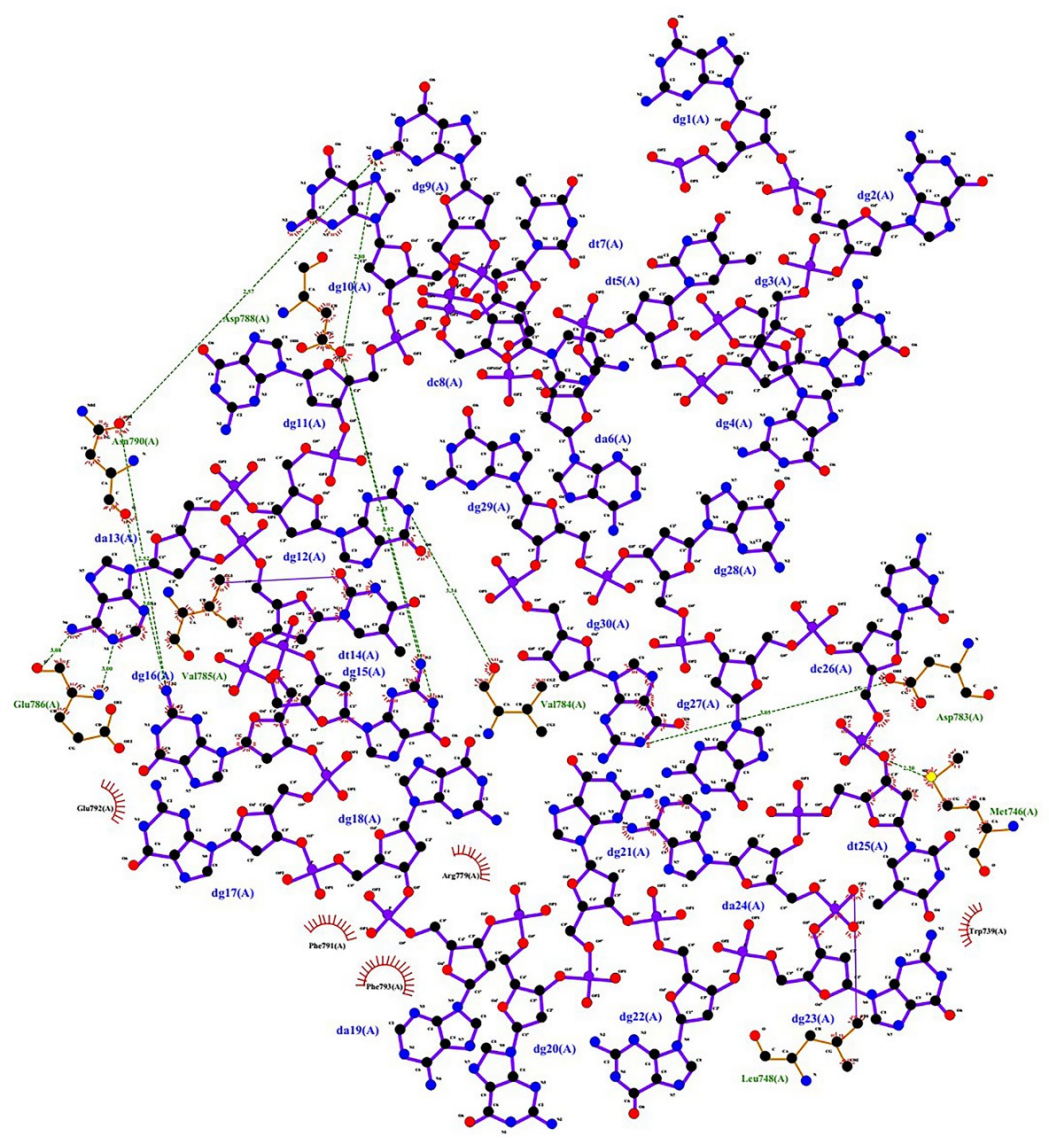
2D diagram of Apt31-E2 protein complex drawn by ligplot. The molecule with purple bonds is the aptamer, and the E2 protein amino acids are shown in brown. Nine hydrogen bonds and two covalent bonds have formed between the Apt31 and protein E2. Also, several amino acids are involved in hydrophobic interactions.

Fig 2 illustrates the binding mode of Apt31 to BVDV-1- E2. Also, the 2D diagram of Apt31 and BVDV-1- E2 interaction is shown in Fig 3. Nine hydrogen bonds and several hydrophobic interactions were detected between Apt31 and BVDV-1 E2 protein. Corresponding protein residues and aptamer nucleotides involved in interactions are shown in Table 3.

**Table 3:** Responsible amino acids and nucleotides for the interaction of Apt31 and E2 protein.

Interaction type	Amino acid	Nucleotide
H-bond	Met746	DT25
	Asp783	DG27
	Val784	DG12
	Asp788	DG15 (×2), DG9
	Asn790	DG9, DG16 (×2)
	Gln786	DA13 (×2)
Van der Waals force	Trp739, Phe 793,	-
	Glu792, Arg779,	
	Phe791	

In recent studies, bioinformatics methods have been widely used for the *de novo* design of aptamers [46–48] or refinement of the aptamers which are selected via SELEX [49, 50]. Morena et al. [47] has utilized *in silico* methods including an elitist genetic algorithm and an RNA inverse process and several web servers for molecular docking analysis (ZDock, HDock, and QVINA-W) to identify three specific aptamers with similar 3D conformations for inhibiting SARS-CoV-2 main protease. Then, they performed molecular dynamics to choose the strongest and the highest stable complex of the aptamer-SARS-CoV-2 main protease. Hosseini et al. [51] designed an ssDNA aptamer via bioinformatics tools and then employed that aptamer in conjugation with silver nanoparticles for inhibition of biofilm formation on the surface of titanium implants. In their study, UNAFold and H-DOCK online servers were used for secondary structure prediction and molecular docking analysis, respectively. In addition, in 2021, an *in silico* study was done by our group to design a G-quadruplex aptamer for specifically reacting to the receptor-binding domain (RBD) region of the spike protein of SARS-CoV-2 [52]. Since G-quadruplex structures are complicated and not all of the structure-prediction web servers and programs can deal with them, *in silico* design and analysis of G-quadruplex aptamers is more challenging. To the best of our knowledge, in the current study, for the first time, in addition to de novo designing a G-quadruplex aptamer for binding to a specific domain of BVDV E2 protein, its binding, and specificity were validated experimentally.

### 3.2. Real-time PCR analysis and BVDV copy number measurement

The viral copy number within the plasma was quantified utilizing the standard curve of BVDV-1 (S1 Fig), which was established in the laboratory of the Biotechnology Department at the University of Isfahan, Iran, and was generated using Real-Time PCR Systems (StepOne^™^, Applied Biosystems, US). The result of qPCR, as the gold standard technique for the detection of BVDV, validated the BVDV infection of the prepared samples with 7.2×107–5.5×10^11^ virus copies/ml (S2 Fig). According to the reported standard curve for BVDV in literature, the least concentration of the virus which can be amplified by the qPCR technique is less than 10 copies/ml [53].

### 3.3. Fabrication of aptasensor

#### 3.3.1. Gold nanoparticle synthesis and characterization

A citrate reduction reaction was used to synthesize gold nanoparticles. The AuNPs exhibited a strong absorption band at 520 nm indicating the appropriate small size of particles. According to the literature, AuNPs with a size up to 50 nm shows a maximum absorption peak at 520–530 nm wavelength, in spectrophotometry, while the peak has a gradual red shift along with the changes in nanoparticle size [54]. DLS measurement showed AuNPs with an average size of 31.7 nm (S4 Fig) and spherical morphology of AuNPs was detected in TEM image (S3 Fig). Zeta potential analysis of AuNPs indicates that the surface of the nanoparticles was covered adequately with the negative charge citrate groups (-41.8 mV) (S4B Fig) leading to the stability of AuNP colloid.

### 3.4. Aptamer G-quadruplex structure forming analysis

**Fig 4:**
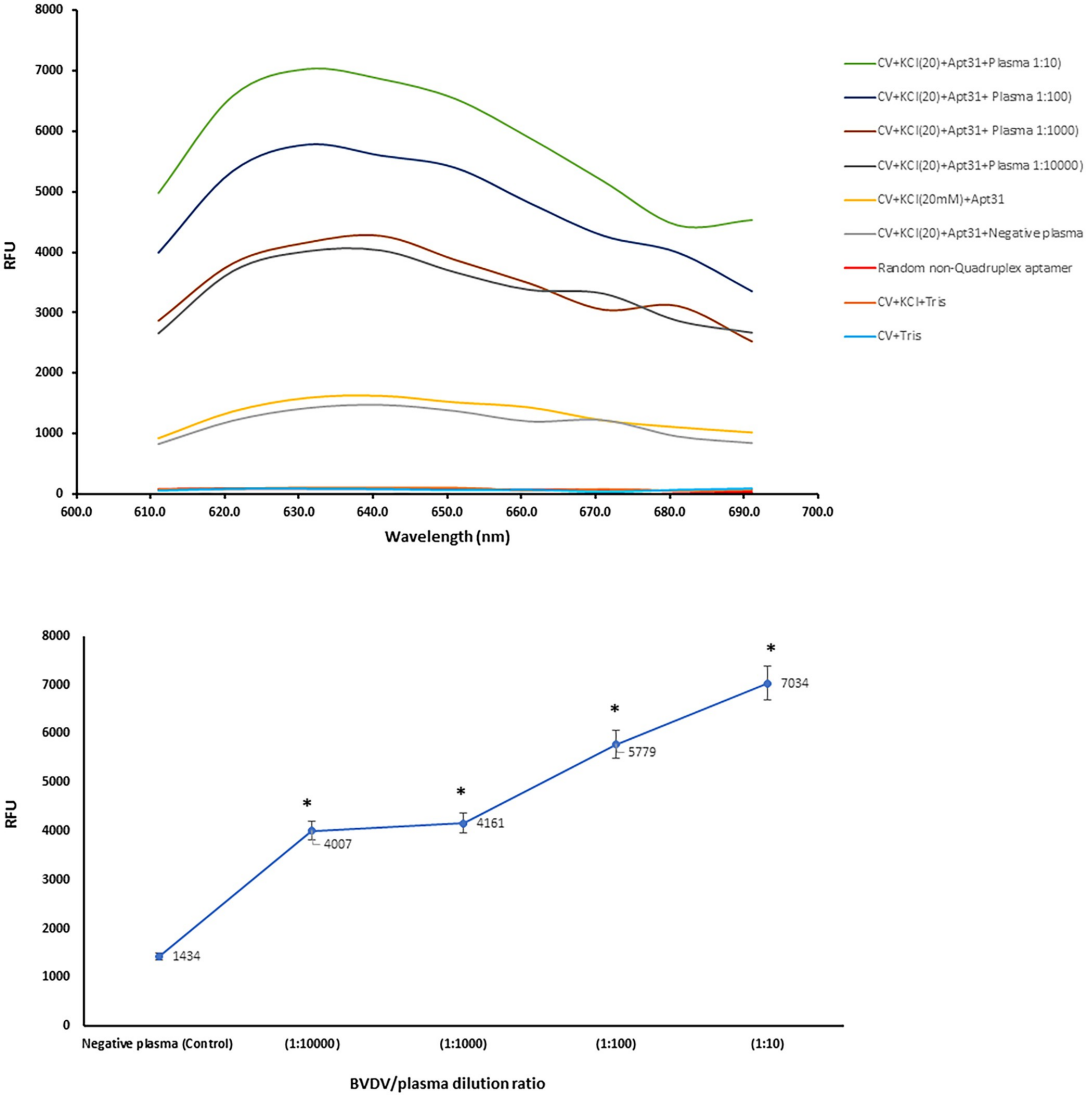
Fluorescence intensity of the crystal violet (CV) in the presence of the Apt31 and BVDV. The fluorescence changes in relative fluorescence units (RFU) for crystal violet dye against a) emission wavelengths (600–700 nm) in the presence of KCl, aptamer, and various dilutions of BVDV-infected plasmas: BVDV-negative plasma, 1:10, 1:100, 1:1000 and 1:10000. (b) various dilutions of BVDV-infected plasma at the emission wavelength of 631 nm. The experimental conditions: 0.25 μM CV, 0.125 μM aptamer, 1 mM KCl, 20 mM Tris–HCl buffer (pH 7.0), excitation wavelength: 580 nm, emission wavelength range:600–700 nm. Values with *p* < 0.05 (*) were considered statistically important.

To determine the G-quadruplex structure of the aptamer, the fluorescence intensity of the crystal violet in presence of the Apt31 was measured. The crystal violet, a triphenylmethane dye, has fluorescence excitation and emission wavelength of 540 nm and 640 nm, respectively in the presence of G-quadruplex DNA structures [55]. It is widely known that CV exhibits a significantly enhanced fluorescence intensity upon double or single-stranded G-quadruplex DNA oligonucleotides [56]. As shown in Fig 4, the fluorescence of free CV dye with and without KCl was very weak, but when aptamer solution was added, the relative fluorescence units (RFU) significantly increased to ~1700. The reason is that CV is intercalated between stacked guanine tetrads and becomes fluorescently active [55, 57]. This change in the fluorescence intensity of CV approves the G-quadruplex structure of the Apt31 in the presence of KCl. Furthermore, adding different dilutions of BVDV-infected plasma to the Apt31-CV solution could dramatically enhance RFU from ~1700 to 4000–7000 depending on the concentration of BVDV. While BVDV-negative (normal) plasma showed no change in fluorescence intensity. The results confirmed that Apt31 binds to E2 protein with a G-quadruplex structure, and the presence of the E2 can stabilize and even induce the quadruplex structure of the aptamer. There are several reports confirming fluorescence intensity changes of CV in presence of G-quadruplex aptamers and their ligands as a technique for assaying the specificity of the aptamer for its ligand [56, 58, 59]. In the study of Cheng *et al*. [60], the addition of saxitoxin as the specific ligand of M-30f aptamer led to the intensification of CV fluorescence, although in Dai *et al*. [61] report, CV fluorescence intensity decreased after the addition of tetracycline (ligand) into the aptamer solution. Accordingly, a change in fluorescence intensity of a fluorescent dye, regardless of increase or decrease, is an index for determining the specificity of an aptamer for its ligand.

### 3.5. Gold nanoparticles salt tolerance test and optimization of aptamer concentration

**Fig 5:**
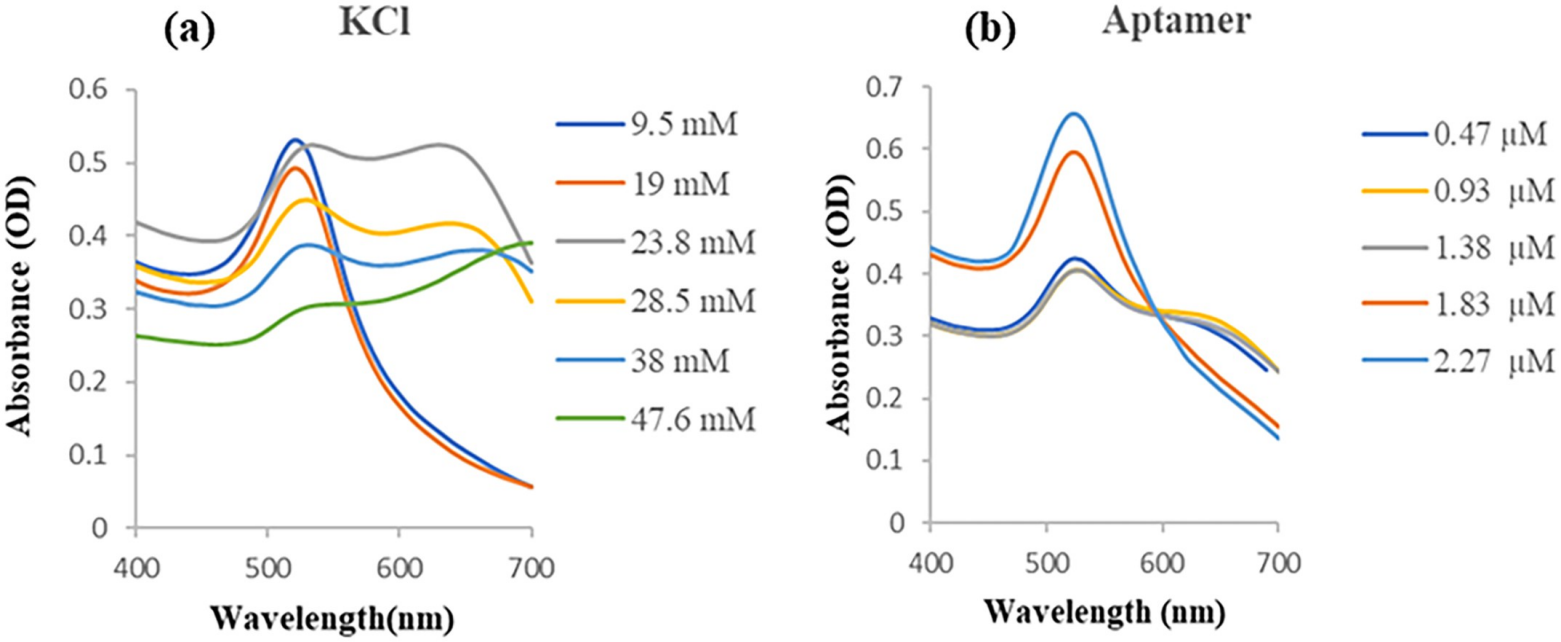
The spectroscopy of gold nanoparticles salt tolerance test. The UV-Vis absorbance spectra for AuNPs in 400–700 nm wavelength in the presence of (a) final KCl concentrations of 9.5 mM to 47.6 mM, (b) different concentrations of aptamer and 23.8 mM KCl.

In the salt-induced aggregation test of AuNPs, different concentrations of KCl were used. As shown in Fig 5a, the AuNP colloid was resistant to added KCl solution with final concentrations of 9.5 and 19mM, but was sensitive to 23.8 mM or higher final concentrations. Aggregation of nanoparticles as a result of sensitivity to the salt concentration led to a red shift in the absorbance peak. The concentration of 500 mM was selected as the minimum concentration of KCl salt which can visually change the AuNP solution color from red to purple.

According to the absorbance spectra in Fig 5b, the optimum concentration of aptamer which can inhibit aggregation of AuNPs was 1.83 μM. In this value, aptamer DNA molecules absorb thoroughly into the negative charge citrate groups on the surface of AuNPs by their positive groups (bases). The negative groups of aptamer DNA molecules expose to the surface and make repulsion between nanoparticles [62]. In this state, the AuNPs become resistant to the 23.8 mM concentration of KCl added to the solution. The 1.83 μM final concentration of aptamer was chosen as the minimum concentration of aptamer which can visually inhibit the AuNP solution color change from red to purple in the presence of KCl. The result of the DLS instrument showed that the hydrodynamic radius of non-functionalized AuNPs from a mean size of around 31 nm increased to around 36 nm after the binding of aptamer on the surface of the AuNPs (S4A Fig). In addition, the conjugation of the aptamer could make the surface charge of the AuNPs more positive, -35.2 mV (S4B Fig).

FTIR spectra of non-functionalized AuNPs in comparison to functionalized AuNPs are shown in S5 Fig. The bands in the range of 3200–3500 cm^-1^ are attributed to N-H stretching of primary amines. There is a band around 3423.03 cm-1 in both AuNP and AuNP-aptamer spectra. A decrease in the intensity of this band indicates that hydrogen bonding is forming between N-H groups of aptamer and AuNPs surface. Hydrogen bonding as a strong dipole-dipole interaction can affect the strength and position of the N-H stretching vibration. Furthermore, there is a red-shift in this peak indicating that the N-H bond is becoming weaker or that the hydrogen bond formation is occurring. Also, changes in electron density or polarization of the amine group (electronic effect) and Changes in molecular geometry due to interactions with other molecules or surfaces (steric effect) can result in the vibrational frequency. The band at 1585.2 cm^-1^ is commonly assigned to the aromatic ring vibration or aromatic C = C stretching vibration. The intensity increase in this peak in aptamer-coated AuNPs may be caused by the adsorption of the aptamer onto the gold nanoparticle surface. This adsorption can make changes in the local environment and electronic properties of aromatic rings. Furthermore, an increase in the intensity of the peak at 1395.25 cm^-1^, which is attributed to bending vibrations of methyl (CH_3_) groups of the aptamer, compared to AuNP spectra, affirms the aptamer coating of AuNPs.

### 3.6. Aptasensor sensitivity and specificity analysis

**Fig 6:**
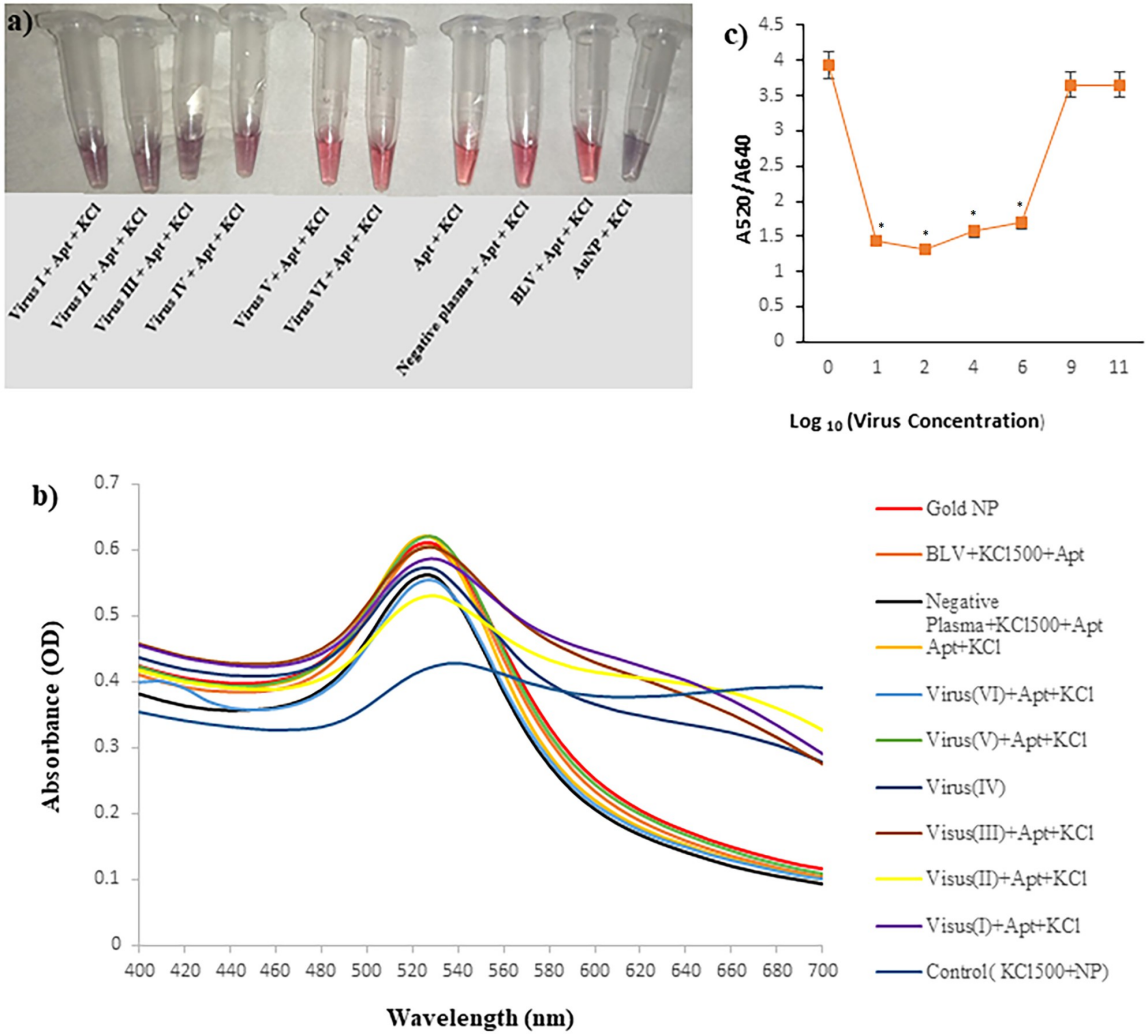
Color change analysis of the aptasensor. (a) visual color change of AuNPs from red to purple in the presence of aptamer, KCl, and different concentrations of the BVDV from 0.27 to 2.7×10^10^ copies/ml, (b) the absorbance spectra of the samples at 400–700 nm wavelength. (c) The corresponding plot of A520/A640 against log10 virus concentration (copies/ml) from I to VI. (I): 0.27, (II): 2.7×10^1^, (III): 2.7×10^3^, (IV): 2.7×10^5^, (V): 2.7×10^8^, (VI): 2.7×10^10^ copies/ml. Apt: Apt31, BLV: Bovine leukemia virus. Values with *p* < 0.05 (*) were considered statistically important.

The sensitivity and specificity of the designed Apt31-AuNP system were assayed in the presence of the BVDV-infected plasma dilutions and BLV, respectively. As it is shown in Fig 6a, adding 10 μl of 500mM KCl into the vials containing 0.27 to 2.7×10^10^ copy numbers of the BVDV (KCl final concentration of 23.8 mM) leads to aggregation of AuNPs solution and subsequently a color change of red to purple occurred. However, no color change was observed for vials containing aptamer, aptamer + negative plasma, and aptamer + BLV. Furthermore, in samples containing high copy numbers of the BVDV (2.7×10^8^ and 2.7×10^10^ copies/ml^)^, the solution color remained red. The reason is that BVDV particles can interfere with AuNP aggregation. It is thought to be related to the interactions between the virus and the AuNP surface. In several studies, it has been observed that a higher concentration of virus can protect the surface of the AuNPs from salt-induced aggregation [63, 64], probably with the same mechanism that proteins do for stabilizing the AuNP surface. The results indicated that the designed AuNPs-aptamer system can detect very low copy numbers of the BVDV (0.27 copies/ml). On the other hand, clinical plasma samples should be diluted to eliminate virus-interfering effects and the virus be detectable via the color change of the solution. In Fig 6b, the UV-vis absorbance spectra of the solutions include a) AuNPs (control), b) AuNPs+ Apt31, c) AuNPs+ Apt31+Negative plasma, d) AuNPs+ Apt31+BLV, e-j) BVDV copy number from 0.27 to 2.7×10^10^ after addition of 23.8 mM KCl solution is drawn. As mentioned above, AuNPs have a maximum peak at the wavelength 520–530 nm. In these spectra, a red shift is observed in AuNPs+Apt31+BVDV and control vials because of AuNPs aggregation. Fig 6c shows the A520/A640 values against varying virus concentrations. Sine in these particular sensing methodologies the color change takes place abruptly in a certain concentration of salt and target virus (not linearly proportional to the increase in the concentration of the virus), thereby resulting in a non-linear curve. Moreover, in our experimental assessments, extremely elevated virus concentrations had an impact on the aggregation of AuNP (as elaborated above). This occurrence contributed to the distinctive U-shaped curve pattern.

In addition, field emission scanning electron microscope (FESEM) characterization images of three samples: a) AuNPs, b) AuNPs+Apt31+KCl, and c) AuNPs+Apt31+BVDV+KCl approved the aggregation of AuNPs in presence of BVDV (S6 Fig).

The application of gold nanoparticles in the development of biosensors has garnered significant attention in recent times [65], especially non-modified aptamer-AuNP biosensors due to their simplicity, high sensitivity, and visibility even by the naked eye.

**Fig 7:**
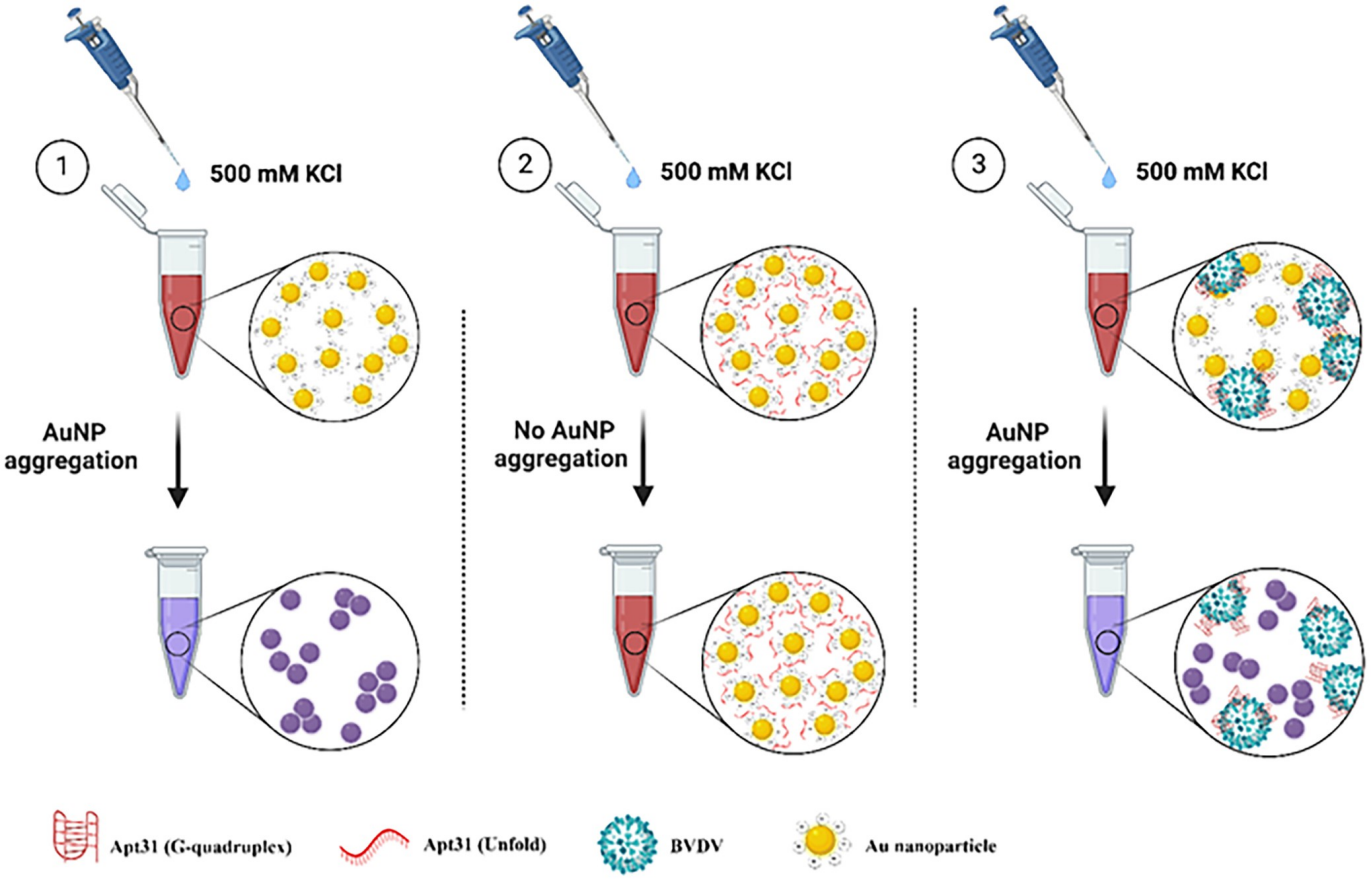
The mechanism of the action of the developed Apt31-biosensor. In stage 1, adding 10 μl of 500mM KCl leads to aggregation of AuNPs, in stage 2, the optimum concentration of Apt31 electrostatically binds to AuNPs and inhibits aggregation of AuNPs after the addition of the KCl solution. In stage 3, if the added plasma sample is infected with BVDV, Apt31 binds to the virus and separates from the surface of AuNPs. Subsequently, the addition of the KCl can result in aggregation of AuNPs and purple-blue color.

The mechanism behind this system is as follows (Fig 7): the normal color of AuNP colloid is red wine, but in the presence of an adequate concentration of salt, the AuNPs stability destroys due to the screening surface charge. Thus, a color change from red to blue occurs. With the addition of aptamer solution to the AuNP colloid, the needed concentration of salt to induce AuNPs aggregation increases. Positive N atoms of DNA aptamer interacts with AuNPs, while negative groups such as phosphates tend to outside, leading to retaining a negative charge of AuNPs surface and tolerance of colloid to the salt. In the presence of the target virus (no other viruses), the affinity of aptamers to the virus is high and can overcome electrostatic interaction between aptamer and AuNPs. As a result of the desorption of aptamer from the AuNPs surface, AuNP aggregation occurs after salt addition. For more in-depth information see [66–69]. Recently, an aptasensor has been developed by Etedali et al. [62] for the rapid detection of the white spot syndrome virus in shrimp. They used the aptamer-AuNP-based colorimetric technique and detected the virus at as low as 10^4^ copies/ml. In addition, the used aptamer in their work was designed via bioinformatics tools. Lerga et al. [70] utilized an aptamer-AuNP system similar to our work for the determination of histamine in foodstuffs. Their biosensor showed a good correlation with the results of HPLC and ELISA kit as standard methods for the measurement of histamine. A different AuNP aggregation-induced system has been used by Sharief et al. [71] for point-of-use food contamination testing. In their study, the utilization of carbohydrate-coated nanoparticles has demonstrated tremendous potential in identifying and capturing pathogens from food matrices in under seven hours.

In 2014, Jee-Woong Park et al. [72] reported an aptamer pair (reporting and capturing aptamers) for the detection of BVDV-1 using the immobilization-free SELEX method. Then, they used the screened 66 nucleotide aptamers for the fabrication of an aptasensor based on the immobilization of aptamers on the gold nanoparticle and SPR gold chip. The comparison of the designed aptamer with their aptamers shows that Apt31 1) has a more stable structure because of forming G-quadruplex, 2) has lower synthesis costs due to a lower number of nucleotides, 3) is more efficient in detecting BVDV-1 (the designed biosensor LOD of 29.7 copies/ml vs. Park et al. biosensor LOD of 800 copies/ml), and 4) is designed for specific binding to the conserved region of the E2 protein of BVDV (not a whole virus). The comparison of our developed aptasensor with previously reported detection methods are summarized in Table 4.

**Table 4:** Comparison of the present method with the recently reported methods for the detection of BVDV.

Analytical method	Detected entity	Sample Type	Limit of detection	Reaction time	Reference
TaqMan real-time PCR (RTm-PCR)	Viral RNA	Feces	1.55 copies/μL	~180 min	[73]
Electrochemical detection with BP@AuNP	Virion	Standard virus sample	0.259 (copies/mL)	10 min	[74]
Dual RT-RPA LFD assay^1^	viral RNA	Standard and clinical samples	50 RNA molecules	30 min	[75]
Dot-blotting method	Virion	Standard virus sample	4.4 copies/mL	~ 60 min	[76]
Aptasensor (SPR gold chip)	Virion	Standard virus sample	800 copies/ml	~ 40 min	[72]
Aptamer-based Biosensor	Virion	Clinical Plasma Samples	0.27 copies/ml	20 min	This study

^1^ reverse transcription recombinase polymerase amplification (RT-RPA) and lateral flow dipstick (LFD)

It is generally presumed that PI animals are antibody-negative [77]. Therefore, antigen-based tests are more efficient in PI diagnosis. The test platforms available for the diagnosis of PIs include virus isolation, antigen-based ELISA, and PCR [13]. Most of these techniques are time-consuming. Furthermore, the RNA genome of the virus is not stable for a long time. Subsequently, in PCR-based methods, the transfer of samples to the specialized laboratory leads to false negative reports in some cases. The developed aptasensor is not only antigen-based and can early diagnose PIs, but also can be used on a farm without the need for complex equipment.

### 3.7. Sample testing and data analysis

Among fifty plasma samples assessed through our developed aptasensor, three samples detected false positive, while all BVDV-infected samples truly identified positive. As a result, the accuracy and precision of the aptasensor achieved, 94% and 90%, respectively.

## 4. Conclusion

In this study, we have achieved a notable milestone by creating a colorimetric biosensor that utilizes a G-quadruplex aptamer-AuNP system to identify the presence of BVDV-1. Detection of very low copies of the virus was achieved with the designed G-quadruplex aptamer and AuNP-aptamer system (0.27 copies/ml). Furthermore, some advantages such as simplicity, naked-eye visualization, low cost, and high stability can make the developed aptasensor a good candidate for a rapid detection method on livestock farms. The aptamer used in the biorecognition element of the biosensor was computationally designed. G-quadruplex forming of the aptamer and its specific binding to BVDV were experimentally confirmed via fluorescent intensity measurement in presence of crystal violet. Our designed G-quadruplex aptamer showed high selectivity and sensitivity for the detection of BVDV-1 indicating the tremendous potential of bioinformatics technique for the selection of specific aptamers. The biosensor experimental validation tests showed outstanding results in comparison to real-time PCR and ELISA as standard tests for the detection of BVDV-1.

## Supporting information

S1 Fig qPCR standard curve for BVDV genotype1. The standard curve illustrates the relationship between cycle threshold (Ct) values and the logarithm of the initial DNA template concentration. Error bars indicate standard deviations.
(TIF)

S2 Fig Amplification of BVDV cDNA. (a) Real-time PCR amplification of BVDV cDNA, three samples with three replicates, positive control (PC) and negative control (without template cDNA) were amplified, (b) Gel electrophoresis analysis of the PCR products of BVDV cDNA amplification (222 bp) on 1% agarose. L: 100-bp ladder, 1,2,3,4: the number of BVDV samples, NC: negative control.
(TIF)

S3 Fig The TEM image of the AuNPs synthesized by the citrate reduction method. (TIF)

S4 Fig Comparison between non-functionalized AuNPs and aptamer-functionalized AuNPs in terms of a) particle size obtained from DLS analysis, and b) zeta potential. (TIF)

S5 Fig FTIR spectra of non-functionalized AuNPs and aptamer-functionalized AuNPs (APT+AuNP). (TIF)

S6 Fig FESEM images of AuNPs in different conditions. (a) AuNPs colloid, (b) AuNPs colloid+ 1.83 μM aptamer + 23.8 mM KCl, (c) aggregated AuNPs + 1.83 μM aptamer + 23.8 mM KCl in the presence of BVDV-infected plasma (2.97×10^5^ copies/ml).
(TIF)

S1 Graphical abstract (TIF)

## References

[1] Walz PH. CHAPTER 24—Bovine Viral Diarrhea Virus. In: Anderson DE, Rings DM, editors. Food Animal Practice (Fifth Edition). Saint Louis: W.B. Saunders; 2009. p. 96–106.

[2] Mungthong K, Khaing ST, Otsubo T, Hatanaka C, Yoneyama S, Hisamatsu S, et al. Broad detection and quick differentiation of bovine viral diarrhea viruses 1 and 2 by a reverse transcription loop-mediated isothermal amplification test. Journal of Veterinary Medical Science. 2021:83(8):1321–9. Epub 2021/06/25. doi: 10.1292/jvms.20-0742 . 34162783 PMC8437728

[3] Chi S, Chen S, Jia W, He Y, Ren L, Wang X. Non-structural proteins of bovine viral diarrhea virus. Virus Genes. 2022:1–10. doi: 10.1007/s11262-022-01914-8 35614328 PMC9131992

[4] Wang L, Wu X, Wang C, Song C, Bao J, Du J. Origin and transmission of bovine viral diarrhea virus type 1 in China revealed by phylodynamic analysis. Research in veterinary science. 2020;128:162–9. doi: 10.1016/j.rvsc.2019.11.015 31809973

[5] de Oliveira Freitas C, de Oliveira PSB, Monteiro FL, Noll JCG, Silva Júnior JVJ, Weiblen R, et al. Sequence analysis of the DA domain of glycoprotein E2 of pestiviruses isolated from beef cattle in Southern Brazil. Archives of Virology. 2021;166(4):1163–70. doi: 10.1007/s00705-020-04910-1 33554289

[6] Li T, Huang M, Xiao H, Zhang G, Ding J, Wu P, et al. Selection and characterization of specific nanobody against bovine virus diarrhea virus (BVDV) E2 protein. PLOS ONE. 2017;12(6):e0178469. doi: 10.1371/journal.pone.0178469 28582444 PMC5459339

[7] Wang F-I, Deng M-C, Huang Y-L, Chang C-Y. Structures and functions of pestivirus glycoproteins: Not simply surface matters. Viruses. 2015;7(7):3506–29. doi: 10.3390/v7072783 26131960 PMC4517112

[8] Goto Y, Yaegashi G, Fukunari K, Suzuki T. An Importance of Long-Term Clinical Analysis to Accurately Diagnose Calves Persistently and Acutely Infected by Bovine Viral Diarrhea Virus 2. Viruses. 2021;13(12):2431. doi: 10.3390/v13122431 34960700 PMC8705094

[9] Klimowicz-Bodys MD, Polak MP, Płoneczka-Janeczko K, Bagnicka E, Zbroja D, Rypuła K. Lack of Fetal Protection against Bovine Viral Diarrhea Virus in a Vaccinated Heifer. Viruses. 2022;14(2):311. doi: 10.3390/v14020311 35215904 PMC8879756

[10] Su A, Fu Y, Meens J, Yang W, Meng F, Herrler G, et al. Infection of polarized bovine respiratory epithelial cells by bovine viral diarrhea virus (BVDV). Virulence. 2021;12(1):177–87. doi: 10.1080/21505594.2020.1854539 33300445 PMC7801128

[11] Diao N-C, Chen Z-Y, Shi J-F, Wang Q, Sheng C-Y, Ma B-Y, et al. Prevalence of bovine viral diarrhea virus in ovine and caprine flocks: A global systematic review and meta-analysis. Frontiers in veterinary science. 2021;8. doi: 10.3389/fvets.2021.703105 34869710 PMC8639873

[12] Yarnall MJ, Thrusfield MV. Engaging veterinarians and farmers in eradicating bovine viral diarrhoea: a systematic review of economic impact. Veterinary Record. 2017;181(13):347-. doi: 10.1136/vr.104370 28851755 PMC5738591

[13] Nugroho W, Silitonga RJP, Reichel MP, Irianingsih SH, Wicaksono MS. The Epidemiology and Control of Bovine Viral Diarrhoea Virus in Tropical Indonesian Cattle. Pathogens. 2022;11(2):215. doi: 10.3390/pathogens11020215 35215158 PMC8878523

[14] Zirra-Shallangwa B, González Gordon L, Hernandez-Castro LE, Cook EAJ, Bronsvoort BMdC, Kelly RF. The Epidemiology of Bovine Viral Diarrhea Virus in Low- and Middle-Income Countries: A Systematic Review and Meta-Analysis. Frontiers in Veterinary Science. 2022;9. doi: 10.3389/fvets.2022.947515 36032291 PMC9404877

[15] Moennig V, Becher P. Control of bovine viral diarrhea. Pathogens. 2018;7(1):29. doi: 10.3390/pathogens7010029 29518049 PMC5874755

[16] Hilbe M, Stalder H, Peterhans E, Haessig M, Nussbaumer M, Egli C, et al. Comparison of five diagnostic methods for detecting bovine viral diarrhea virus infection in calves. Journal of Veterinary Diagnostic Investigation. 2007;19(1):28–34. doi: 10.1177/104063870701900105 17459829

[17] Kim JG, Baek SH, Kim S, Kim HI, Lee SW, Phan LMT, et al. Rapid discriminative detection of dengue viruses via loop mediated isothermal amplification. Talanta. 2018;190:391–6. Epub 2018/09/03. doi: 10.1016/j.talanta.2018.08.019 . 30172524

[18] Surti PV, Kim MW, Phan LMT, Kailasa SK, Mungray AK, Park JP, et al. Progress on dot-blot assay as a promising analytical tool: Detection from molecules to cells. TrAC Trends in Analytical Chemistry. 2022;157:116736. doi: 10.1016/j.trac.2022.116736

[19] Xu J, Wu S. Chapter 4—Other Nanomaterials. In: Li G, editor. Nano-Inspired Biosensors for Protein Assay with Clinical Applications: Elsevier; 2019. p. 91–111.

[20] Liu B, Zhuang J, Wei G. Recent advances in the design of colorimetric sensors for environmental monitoring. Environmental Science: Nano. 2020;7(8):2195–213. doi: 10.1039/D0EN00449A

[21] Chambers J, Arulanandam B, Matta L, Weis A, Valdes J. Biosensor recognition elements. Current issues in molecular biology. 2008;10:1–12. 18525101

[22] Tombelli S, Minunni M, Mascini M. Analytical Applications of Aptamers. Biosensors & bioelectronics. 2005;20:2424–34. doi: 10.1016/j.bios.2004.11.006 15854817

[23] Zhao L, Huang Y, Dong Y, Han X, Wang S, Liang X. Aptamers and Aptasensors for Highly Specific Recognition and Sensitive Detection of Marine Biotoxins: Recent Advances and Perspectives. Toxins. 2018;10(11):427. doi: 10.3390/toxins10110427 30366456 PMC6265707

[24] Roxo C, Kotkowiak W, Pasternak A. G-Quadruplex-Forming Aptamers-Characteristics, Applications, and Perspectives. Molecules. 2019;24(20). Epub 2019/10/24. doi: 10.3390/molecules24203781 . 31640176 PMC6832456

[25] Ahirwar R, Nahar S, Aggarwal S, Ramachandran S, Maiti S, Nahar P. In silico selection of an aptamer to estrogen receptor alpha using computational docking employing estrogen response elements as aptamer-alike molecules. Sci Rep. 2016;6:21285-. doi: 10.1038/srep21285 . 26899418 PMC4761961

[26] Kinghorn AB, Fraser LA, Lang S, Shiu SC-C, Tanner JA. Aptamer Bioinformatics. Int J Mol Sci. 2017;18(12):2516. doi: 10.3390/ijms18122516 . 29186809 PMC5751119

[27] Zhang W, Liu QX, Guo ZH, Lin JS. Practical Application of Aptamer-Based Biosensors in Detection of Low Molecular Weight Pollutants in Water Sources. Molecules. 2018;23(2):344. doi: 10.3390/molecules23020344 . 29414854 PMC6017897

[28] Song K-M, Lee S, Ban C. Aptamers and their biological applications. Sensors (Basel). 2012;12(1):612–31. Epub 01/09. doi: 10.3390/s120100612 . 22368488 PMC3279232

[29] Zhu L, Zhao Y, Yao S, Xu M, Yin L, Zhai X, et al. A colorimetric aptasensor for the simple and rapid detection of human papillomavirus type 16 L1 proteins. Analyst. 2021;146(8):2712–7. Epub 2021/03/11. doi: 10.1039/d1an00251a . 33688885

[30] El Omari K, Iourin O, Harlos K, Grimes JM, Stuart DI. Structure of a pestivirus envelope glycoprotein E2 clarifies its role in cell entry. Cell reports. 2013;3(1):30–5. Epub 2013/01/01. doi: 10.1016/j.celrep.2012.12.001 . 23273918 PMC3607223

[31] Li Y, Wang J, Kanai R, Modis Y. Crystal structure of glycoprotein E2 from bovine viral diarrhea virus. Proceedings of the National Academy of Sciences. 2013;110(17):6805–10. doi: 10.1073/pnas.1300524110 23569276 PMC3637714

[32] Mishra SK, Tawani A, Mishra A, Kumar A. G4IPDB: A database for G-quadruplex structure forming nucleic acid interacting proteins. Sci Rep. 2016;6:38144. Epub 2016/12/03. doi: 10.1038/srep38144 . 27905517 PMC5131279

[33] Kikin O, D’Antonio L, Bagga PS. QGRS Mapper: a web-based server for predicting G-quadruplexes in nucleotide sequences. Nucleic Acids Research. 2006;34(suppl_2):W676–W82. doi: 10.1093/nar/gkl253 16845096 PMC1538864

[34] Sripakdeevong P, Kladwang W, Das R. An enumerative stepwise ansatz enables atomic-accuracy RNA loop modeling. Proceedings of the National Academy of Sciences. 2011;108(51):20573. doi: 10.1073/pnas.1106516108 22143768 PMC3251086

[35] Paramasivan S, Rujan I, Bolton PH. Circular dichroism of quadruplex DNAs: Applications to structure, cation effects and ligand binding. Methods. 2007;43(4):324–31. doi: 10.1016/j.ymeth.2007.02.009 17967702

[36] Das R, Karanicolas J, Baker D. Atomic accuracy in predicting and designing noncanonical RNA structure. Nature Methods. 2010;7(4):291–4. doi: 10.1038/nmeth.1433 20190761 PMC2854559

[37] Lyskov S, Chou F-C, Conchúir SÓ, Der BS, Drew K, Kuroda D, et al. Serverification of molecular modeling applications: the Rosetta Online Server that Includes Everyone (ROSIE). PloS one. 2013;8(5):e63906. doi: 10.1371/journal.pone.0063906 23717507 PMC3661552

[38] Yan Y, Zhang D, Zhou P, Li B, Huang SY. HDOCK: a web server for protein-protein and protein-DNA/RNA docking based on a hybrid strategy. Nucleic acids research. 2017;45(W1):W365–w73. Epub 2017/05/19. doi: 10.1093/nar/gkx407 . 28521030 PMC5793843

[39] Wallace AC, Laskowski RA, Thornton JM. LIGPLOT: a program to generate schematic diagrams of protein-ligand interactions. Protein engineering. 1995;8(2):127–34. Epub 1995/02/01. doi: 10.1093/protein/8.2.127 . 7630882

[40] Turkevich J, Stevenson PC, Hillier J. A study of the nucleation and growth processes in the synthesis of colloidal gold. Discussions of the Faraday Society. 1951;11:55–75.

[41] Mohabatkar H, Rabiei P, Alamdaran M. New Achievements in Bioinformatics Prediction of Post Translational Modification of Proteins. Curr Top Med Chem. 2017;17(21):2381–92. Epub 2017/03/31. doi: 10.2174/1568026617666170328100908 . 28356000

[42] Ledlod S, Areekit S, Santiwatanakul S, Chansiri K. Colorimetric aptasensor for detecting Salmonella spp., Listeria monocytogenes, and Escherichia coli in meat samples. Food Science and Technology International. 2020;26(5):430–43. doi: 10.1177/1082013219899593 . 31948282

[43] Aniță DC, Popa E, Aniță A, Oșlobanu LE, Savuța G. Pestivirus spillover effect: molecular detection of bovine viral diarrhea virus in domestic and feral pigs. Pesquisa Veterinária Brasileira. 2020;40:479–83.

[44] Afanasyeva A, Nagao C, Mizuguchi K. Prediction of the secondary structure of short DNA aptamers. Biophysics and Physicobiology. 2019;16:287–94. doi: 10.2142/biophysico.16.0_287 31984183 PMC6975895

[45] Mirosław P, Polak MP. Variability of E2 protein-coding sequences of bovine viral diarrhea virus in Polish cattle. Virus Genes. 2020;56(4):515–21. Epub 2020/04/18. doi: 10.1007/s11262-020-01756-2 . 32300930 PMC7329765

[46] Torkamanian-Afshar M, Nematzadeh S, Tabarzad M, Najafi A, Lanjanian H, Masoudi-Nejad A. In silico design of novel aptamers utilizing a hybrid method of machine learning and genetic algorithm. Molecular diversity. 2021;25(3):1395–407. doi: 10.1007/s11030-021-10192-9 33554306

[47] Morena F, Argentati C, Tortorella I, Emiliani C, Martino S. De novo ssRNA aptamers against the SARS-CoV-2 main protease: in silico design and molecular dynamics simulation. International journal of molecular sciences. 2021;22(13):6874. doi: 10.3390/ijms22136874 34206794 PMC8267631

[48] Bavi R, Liu Z, Han Z, Zhang H, Gu Y. In silico designed RNA aptamer against epithelial cell adhesion molecule for cancer cell imaging. Biochemical and biophysical research communications. 2019;509(4):937–42. doi: 10.1016/j.bbrc.2019.01.028 30648555

[49] Belinskaia DA, Avdonin PV, Avdonin PP, Jenkins RO, Goncharov NV. Rational in silico design of aptamers for organophosphates based on the example of paraoxon. Computational Biology and Chemistry. 2019;80:452–62. doi: 10.1016/j.compbiolchem.2019.05.004 31170561

[50] Lin YC, Chen WY, Hwu ET, Hu WP. In-Silico Selection of Aptamer Targeting SARS-CoV-2 Spike Protein. Int J Mol Sci. 2022;23(10). Epub 2022/05/29. doi: 10.3390/ijms23105810 . 35628622 PMC9143595

[51] Hosseini B, Behbahani M, Dini G, Mohabatkar H, Keyhanfar M. Investigating the anti-streptococcal biofilm effect of ssDNA aptamer-silver nanoparticles complex on a titanium-based substrate. RSC advances. 2022;12(38):24876–86. doi: 10.1039/d2ra04112j 36276899 PMC9475424

[52] Behbahani M, Mohabatkar H, Hosseini B. In silico design of quadruplex aptamers against the spike protein of SARS-CoV-2. Inform Med Unlocked. 2021;26:100757. Epub 2021/10/20. doi: 10.1016/j.imu.2021.100757 . 34664030 PMC8514331

[53] Hou P, Xu Y, Wang H, He H. Detection of bovine viral diarrhea virus genotype 1 in aerosol by a real time RT-PCR assay. BMC veterinary research. 2020;16(1):1–9. 32295612 10.1186/s12917-020-02330-6 PMC7159024

[54] He Y, L pu, Kong L, Liu Z. A study on the sizes and concentrations of gold nanoparticles by spectra of absorption, resonance Rayleigh scattering and resonance non-linear scattering. Spectrochimica acta Part A, Molecular and biomolecular spectroscopy. 2005;61:2861–6. doi: 10.1016/j.saa.2004.10.035 16165025

[55] Umar MI, Ji D, Chan C-Y, Kwok CK. G-quadruplex-based fluorescent turn-on ligands and aptamers: from development to applications. Molecules. 2019;24(13):2416. doi: 10.3390/molecules24132416 31262059 PMC6650947

[56] Chen Y, Wang J, Zhang Y, Xu L, Gao T, Wang B, et al. Selection and characterization of a DNA aptamer to crystal violet. Photochemical & Photobiological Sciences. 2018;17(6):800–6. doi: 10.1039/c7pp00457e 29770378

[57] Kong DM, Ma YE, Wu J, Shen HX. Discrimination of G-quadruplexes from duplex and single-stranded DNAs with fluorescence and energy-transfer fluorescence spectra of crystal violet. Chemistry–A European Journal. 2009;15(4):901–9. doi: 10.1002/chem.200801441 19053101

[58] Kong D-M, Guo J-H, Yang W, Ma Y-E, Shen H-X. Crystal violet–G-quadruplex complexes as fluorescent sensors for homogeneous detection of potassium ion. Biosensors and Bioelectronics. 2009;25(1):88–93. doi: 10.1016/j.bios.2009.06.002 19559594

[59] Bayraç AT, Acar Y. Label-free G-Quadruplex aptamer and Thioflavin-T based turn-off fluorescent detection of ethanolamine. Dyes and Pigments. 2020;172:107788.

[60] Cheng S, Zheng B, Yao D, Kuai S, Tian J, Liang H, et al. Study of the binding way between saxitoxin and its aptamer and a fluorescent aptasensor for detection of saxitoxin. Spectrochimica Acta Part A: Molecular and Biomolecular Spectroscopy. 2018;204:180–7. doi: 10.1016/j.saa.2018.06.036 29933153

[61] Dai Y, Zhang Y, Liao W, Wang W, Wu L. G-quadruplex specific thioflavin T-based label-free fluorescence aptasensor for rapid detection of tetracycline. Spectrochimica Acta Part A: Molecular and Biomolecular Spectroscopy. 2020;238:118406. doi: 10.1016/j.saa.2020.118406 32387918

[62] Etedali P, Behbahani M, Mohabatkar H, Dini G. Field-usable aptamer-gold nanoparticles-based colorimetric sensor for rapid detection of white spot syndrome virus in shrimp. Aquaculture. 2022;548:737628.

[63] Mi X, Lucier EM, Turpeinen DG, Yeo ELL, Kah JCY, Heldt CL. Mannitol-induced gold nanoparticle aggregation for the ligand-free detection of viral particles. Analyst. 2019;144(18):5486–96. doi: 10.1039/c9an00830f 31386701

[64] Yeo ELL, Chua AJS, Parthasarathy K, Yeo HY, Ng ML, Kah JCY. Understanding aggregation-based assays: nature of protein corona and number of epitopes on antigen matters. RSC Advances. 2015;5(20):14982–93. doi: 10.1039/C4RA12089B

[65] Li C-H, Chan M-H, Chang Y-C, Hsiao M. Gold Nanoparticles as a Biosensor for Cancer Biomarker Determination. Molecules. 2023;28(1):364. doi: 10.3390/molecules28010364 36615558 PMC9822408

[66] Kim Y-J, Kim H-S, Chon J-W, Kim D-H, Hyeon J-Y, Seo K-H. New colorimetric aptasensor for rapid on-site detection of Campylobacter jejuni and Campylobacter coli in chicken carcass samples. Analytica Chimica Acta. 2018;1029:78–85. doi: 10.1016/j.aca.2018.04.059 29907294

[67] Yazdian-Robati R, Hedayati N, Ramezani M, Abnous K, Taghdisi SM. Colorimetric gold nanoparticles-based aptasensors. Nanomedicine Journal. 2018;5(1):1–5.

[68] Sabela M, Balme S, Bechelany M, Janot JM, Bisetty K. A review of gold and silver nanoparticle-based colorimetric sensing assays. Advanced Engineering Materials. 2017;19(12):1700270.

[69] Cheng F, He Y, Xing XJ, Tan DD, Lin Y, Pang DW, et al. A gold nanoparticle-based label free colorimetric aptasensor for adenosine deaminase detection and inhibition assay. Analyst. 2015;140(5):1572–7. Epub 2015/01/20. doi: 10.1039/c4an02070g . 25597304

[70] Lerga TM, Skouridou V, Bermudo MC, Bashammakh AS, El-Shahawi MS, Alyoubi AO, et al. Gold nanoparticle aptamer assay for the determination of histamine in foodstuffs. Microchimica Acta. 2020;187(8):1–9. doi: 10.1007/s00604-020-04414-4 32676707

[71] Sharief SA, Caliskan-Aydogan O, Alocilja E. Carbohydrate-coated magnetic and gold nanoparticles for point-of-use food contamination testing. Biosensors and Bioelectronics: X. 2023;13:100322. doi: 10.1016/j.biosx.2023.100322

[72] Park J-W, J Lee S, Choi E-J, Kim J, Song J-Y, B Gu M. An ultra-sensitive detection of a whole virus using dual aptamers developed by immobilization-free screening. Biosensors and Bioelectronics. 2014;51:324–9. Epub 2013/09/03. doi: 10.1016/j.bios.2013.07.052 . 23994614

[73] Liang H, Geng J, Bai S, Aimuguri A, Gong Z, Feng R, et al. TaqMan real-time PCR for detecting bovine viral diarrhea virus. Pol J Vet Sci. 2019;22(2):405–13. Epub 2019/07/04. doi: 10.24425/pjvs.2019.129300 . 31269348

[74] Kim MW, Lee DY, Cho CH, Park CY, Ghosh S, Hyun MS, et al. Sensitive Detection of BVDV Using Gold Nanoparticle-Modified Few-Layer Black Phosphorus with Affinity Peptide-Based Electrochemical Sensor. ACS Appl Bio Mater. 2023;6(4):1621–8. Epub 2023/03/28. doi: 10.1021/acsabm.3c00045 . 36972355

[75] Yang S, Wang QY, Tan B, Shi PF, Qiao LJ, Li ZJ, et al. A lateral flow dipstick combined with reverse transcription recombinase polymerase amplification for rapid and visual detection of the BVDV and BPIV3. J Virol Methods. 2022;299:114343. Epub 2021/11/04. doi: 10.1016/j.jviromet.2021.114343 . 34728269

[76] Kim MW, Park H-J, Park CY, Kim JH, Cho CH, Phan LMT, et al. Fabrication of a paper strip for facile and rapid detection of bovine viral diarrhea virus via signal enhancement by copper polyhedral nanoshells. RSC Advances. 2020;10(50):29759–64. doi: 10.1039/d0ra03677c 35518256 PMC9056175

[77] McDougall S. Effect of calf age on bovine viral diarrhea virus tests. J Vet Diagn Invest. 2021;33(3):528–37. Epub 2021/03/06. doi: 10.1177/1040638721998821 33666123 PMC8120083

