# Supplementary figures and images for: A label-free G-quadruplex aptamer/gold nanoparticle-based colorimetric biosensor for rapid detection of bovine viral diarrhea virus genotype 1
Source: PLoS One. 2024 Jul 30;19(7):e0293561. doi: 10.1371/journal.pone.0293561 (PMC11288453; doi:10.1371/journal.pone.0293561)

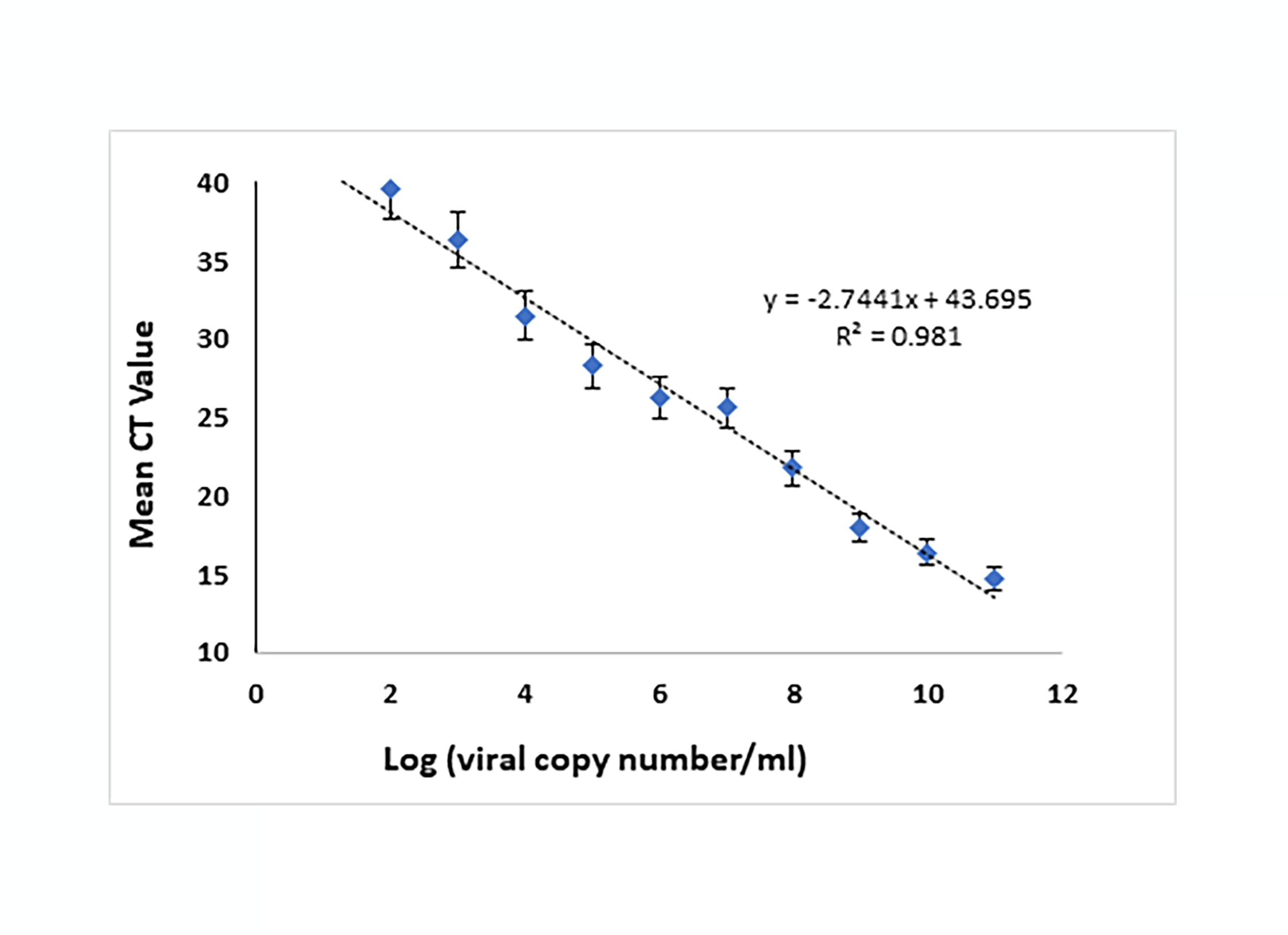

Supplement: S1 Fig — The standard curve illustrates the relationship between cycle threshold (Ct) values and the logarithm of the initial DNA template concentration. Error bars indicate standard deviations. (TIF) [file pone.0293561.s001.tif]

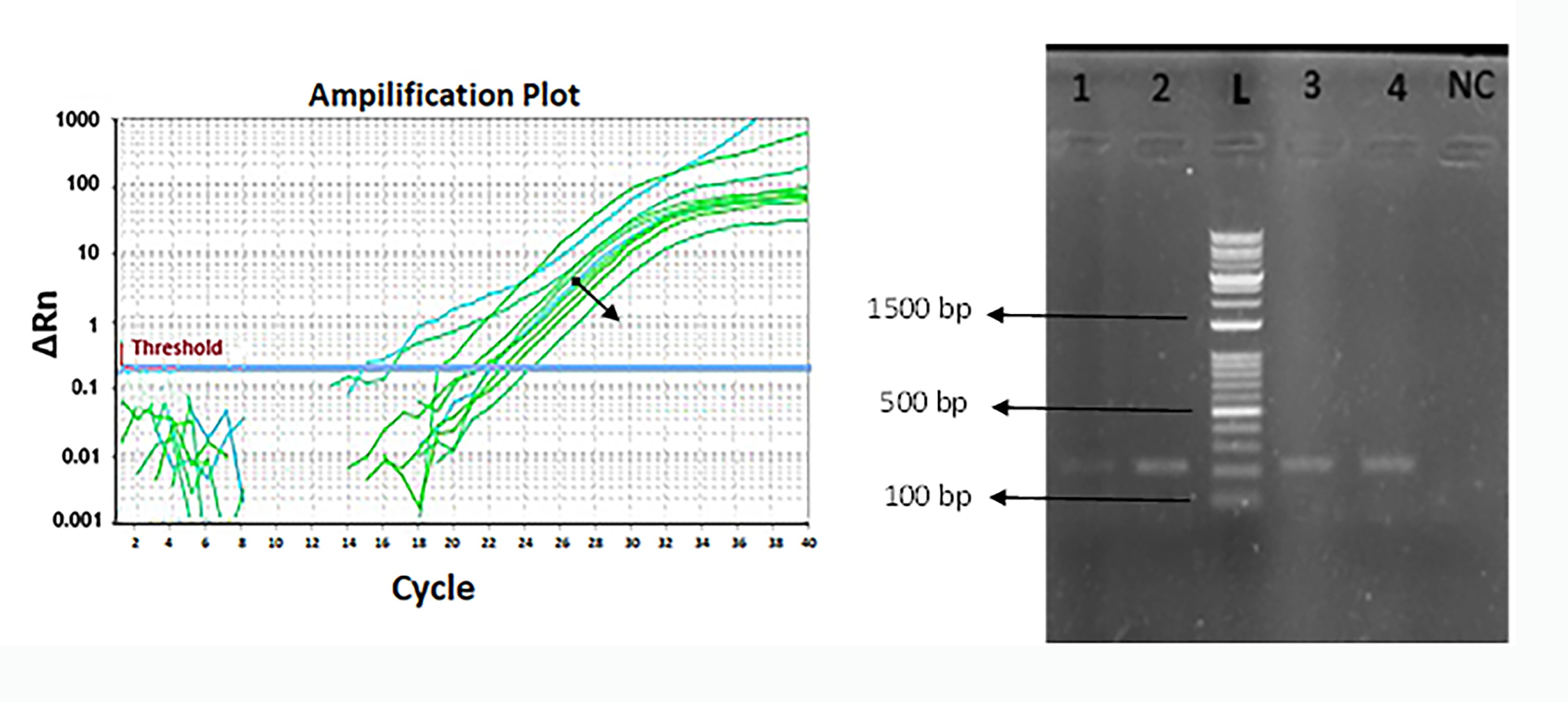

Supplement: S2 Fig — (a) Real-time PCR amplification of BVDV cDNA, three samples with three replicates, positive control (PC) and negative control (without template cDNA) were amplified, (b) Gel electrophoresis analysis of the PCR products of BVDV cDNA amplification (222 bp) on 1% agarose. L: 100-bp ladder, 1,2,3,4: the number of BVDV samples, NC: negative control. (TIF) [file pone.0293561.s002.tif]

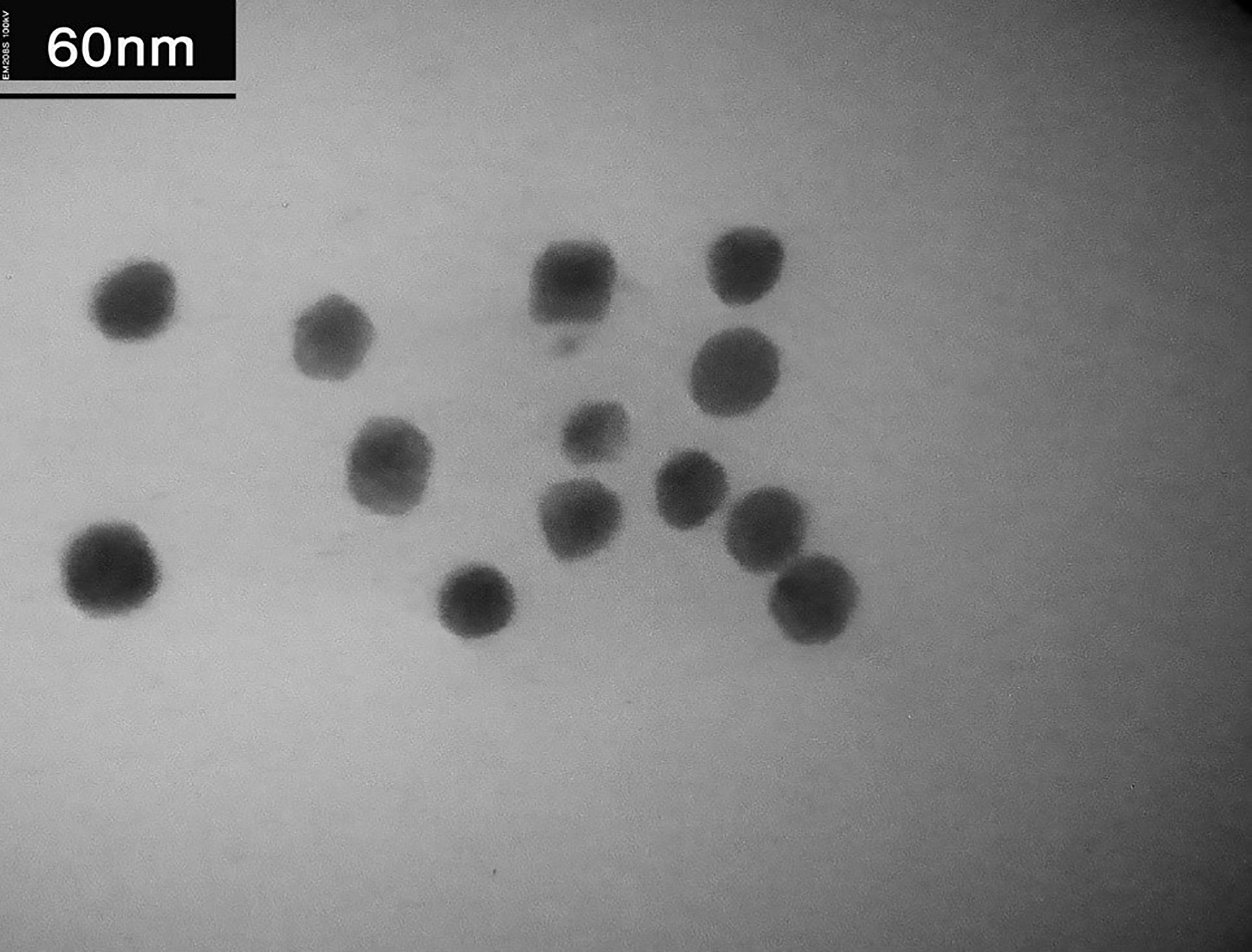

Supplement: S3 Fig — (TIF) [file pone.0293561.s003.tif]

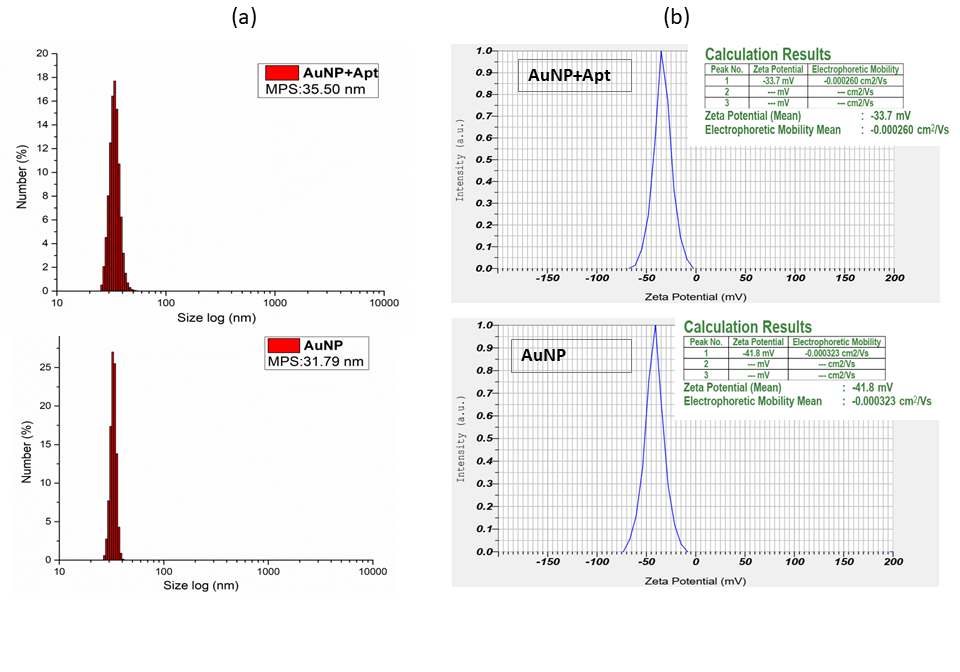

Supplement: S4 Fig — (TIF) [file pone.0293561.s004.tif]

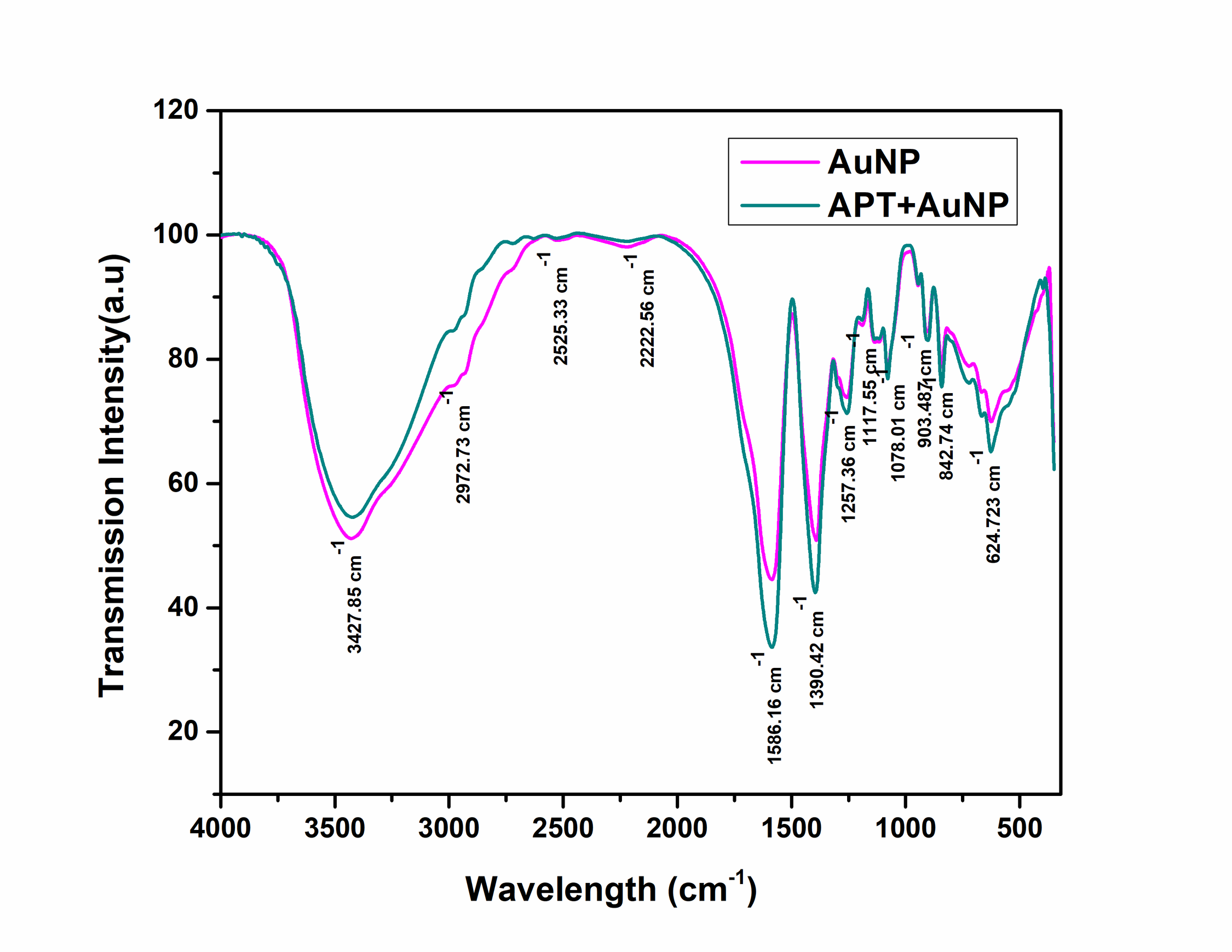

Supplement: S5 Fig — (TIF) [file pone.0293561.s005.tif]

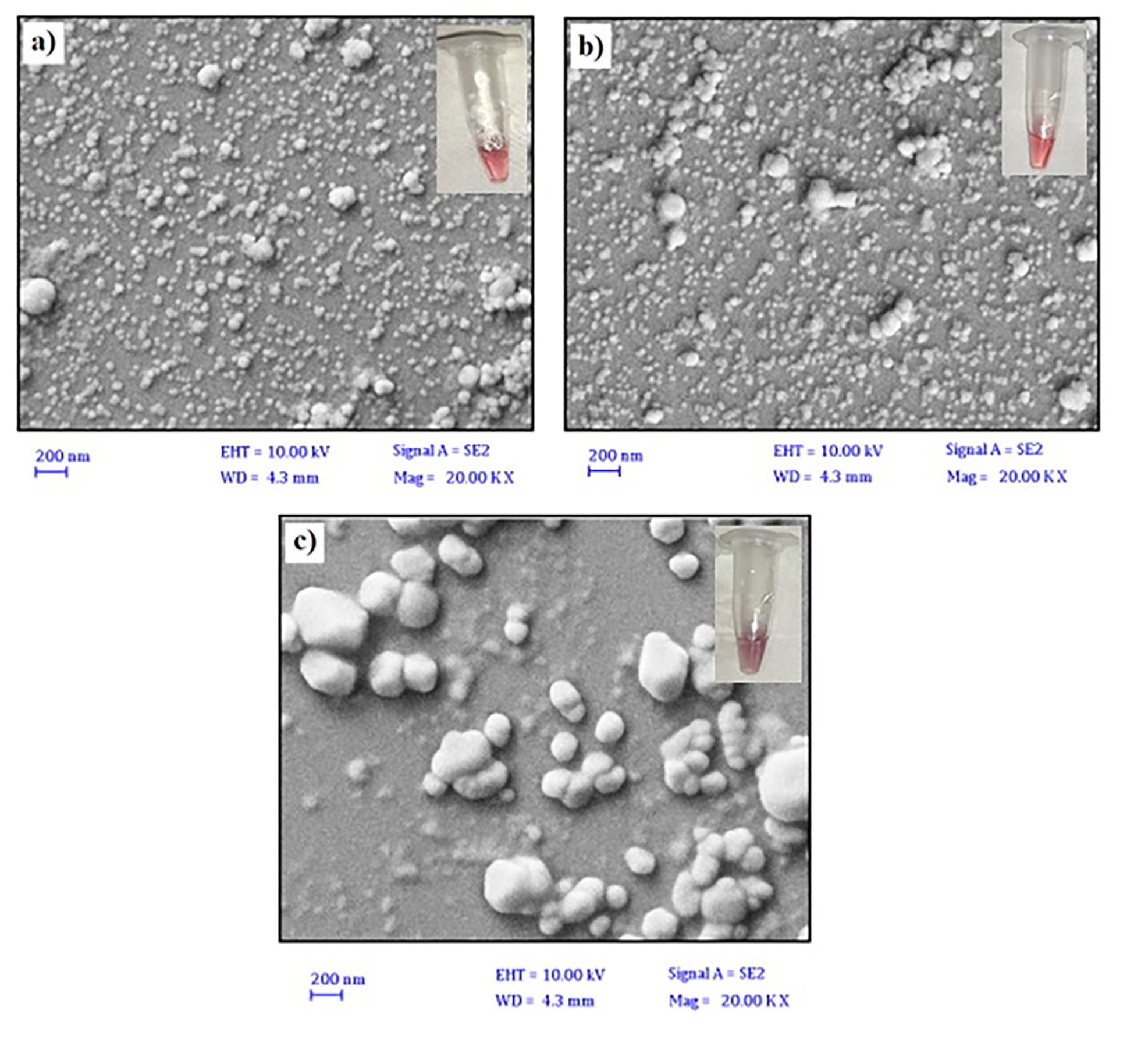

Supplement: S6 Fig — (a) AuNPs colloid, (b) AuNPs colloid+ 1.83 μM aptamer + 23.8 mM KCl, (c) aggregated AuNPs + 1.83 μM aptamer + 23.8 mM KCl in the presence of BVDV-infected plasma (2.97×10^5^ copies/ml). (TIF) [file pone.0293561.s006.tif]

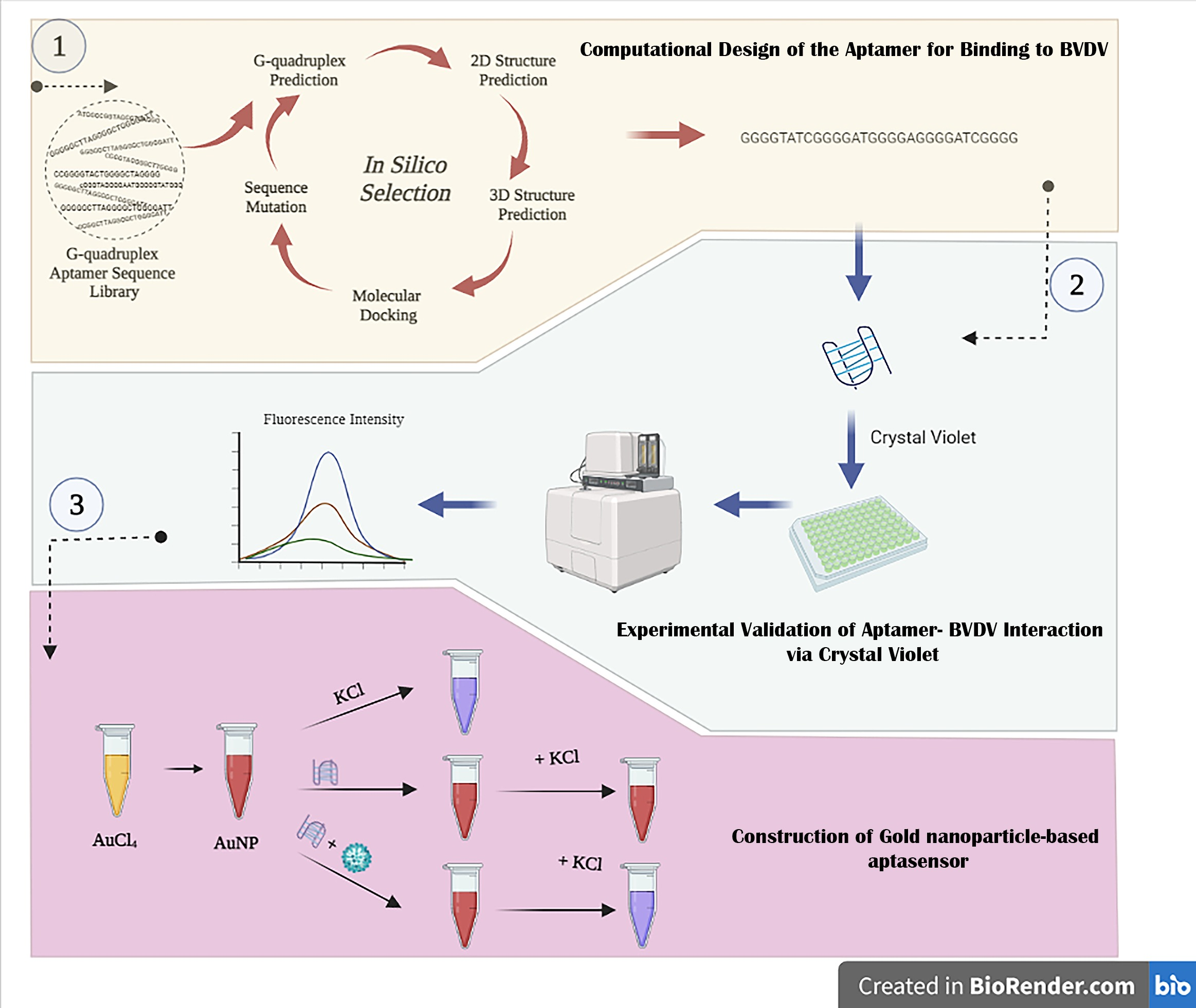

Supplement: S1 Graphical abstract — (TIF) [file pone.0293561.s007.tif]
